# EquiFACS: The Equine Facial Action Coding System

**DOI:** 10.1371/journal.pone.0131738

**Published:** 2015-08-05

**Authors:** Jen Wathan, Anne M. Burrows, Bridget M. Waller, Karen McComb

**Affiliations:** 1 Mammal Communication and Cognition Research, School of Psychology, University of Sussex, Brighton, United Kingdom; 2 Centre for Comparative and Evolutionary Psychology, Department of Psychology, University of Portsmouth, Portsmouth, United Kingdom; 3 Department of Physical Therapy, Duquesne University, Pittsburgh, Pennsylvania, United States of America; ETH Zurich, SWITZERLAND

## Abstract

Although previous studies of horses have investigated their facial expressions in specific contexts, e.g. pain, until now there has been no methodology available that documents all the possible facial movements of the horse and provides a way to record all potential facial configurations. This is essential for an objective description of horse facial expressions across a range of contexts that reflect different emotional states. Facial Action Coding Systems (FACS) provide a systematic methodology of identifying and coding facial expressions on the basis of underlying facial musculature and muscle movement. FACS are anatomically based and document all possible facial movements rather than a configuration of movements associated with a particular situation. Consequently, FACS can be applied as a tool for a wide range of research questions. We developed FACS for the domestic horse (*Equus caballus*) through anatomical investigation of the underlying musculature and subsequent analysis of naturally occurring behaviour captured on high quality video. Discrete facial movements were identified and described in terms of the underlying muscle contractions, in correspondence with previous FACS systems. The reliability of others to be able to learn this system (EquiFACS) and consistently code behavioural sequences was high—and this included people with no previous experience of horses. A wide range of facial movements were identified, including many that are also seen in primates and other domestic animals (dogs and cats). EquiFACS provides a method that can now be used to document the facial movements associated with different social contexts and thus to address questions relevant to understanding social cognition and comparative psychology, as well as informing current veterinary and animal welfare practices.

## Introduction

Facial Action Coding Systems (FACS) provide a method of identifying and recording facial expressions based on the underlying facial (mimetic) musculature and muscle movement [1]. Here we present EquiFACS, a Facial Action Coding System for the domestic horse (*Equus caballus*). Until now, there has been no methodology available that documented all of the facial movements of the horse, allowing a record of all potential facial configurations. There are a number of studies that focus on the facial expressions of horses in one specific context, for example pain [2, 3]. However, EquiFACS provides a comprehensive list of all the facial movements that horses can produce, which can be used to document facial expressions across a wide range of contexts.

### What Are Facial Action Coding Systems?

FACS are objective coding systems for describing facial behaviour. The facial muscles (sometimes known as the mimetic muscles) are a subgroup of muscles innervated by CN7 (the facial nerve). They are characterised by their extensive connections to the superficial fascia and skin of the face, and consequently are responsible for observable changes in the skin (facial movements or expressions) [4]. FACS are frameworks where designated codes (Action Units or AUs) represent the contraction of a particular facial muscle (or set of muscles) and the resulting facial movements [1]. Action Descriptors (ADs) are also used for more general facial movements where the muscular basis either cannot be identified or is the result of a different muscle set (e.g. deep muscles). This creates a reliable system that people can be trained to use and that describes facial actions in a standardised way, avoiding subjective assessments of expression. This is particularly important as the recording and analysis of facial expressions can be subject to a large degree of observer bias and influenced by the perceived emotional context [5–7]. Consequently, frameworks that quantify behaviour and allow reliable, objective measurements are needed.

The original FACS was developed for use in humans [1] and this framework has since been applied to a number of different primates and domestic animals (chimpanzees (*Pan troglodytes*)[8], orangutans (*Pongo pygmaeus*)[9], rhesus macaques (*Macaca mulatta*)[10], *hylobatids* (gibbons and siamangs)[11], dogs (*Canis familiaris*)[12] and cats (*Felis catus*)[13]. This methodology provides compatible systems that allow direct comparisons using identical techniques across species with a different facial morphology (e.g. [14]). EquiFACS is the first attempt to develop this system for an animal with laterally placed eyes and an elongated face.

### Why Are Horses an Interesting Model?

Horses belong to the order Perissodactyla, and within this they are one of nine species in the family Equus. By the end of the Pleistocene era (approx. 2.5 million – 12,000 years ago) wild horses (*Equus ferus*) roamed across Europe, Asia and North America, and it is estimated that the domestication of horses began around 5000 years ago [15]. However, by the middle of the twentieth century horses had become extinct in the wild, yet were thriving as a domestic species [16]. Domestication can dramatically influence the social, cognitive, and morphological characteristics of a species, and the study of domestic species is of great interest from both welfare and evolutionary perspectives [17–19].

Horses are long-lived social animals. Feral populations have demonstrated that without domestic pressures horses would live in a society comprising of several small groups or ‘bands’ that share space and resources, and to which membership stays relatively stable over time. Bands have large, overlapping ranges so horses regularly come into contact with many other conspecifics, and inter-band dominance indicates that within the larger herd established social relationships exist [20]. Consequently, horses show fission-fusion dynamics; a variation of the same complex social organisation that is seen in humans, bonobos, chimpanzees, and macaques, as well as elephants, spotted hyenas and many cetaceans [21]. Group life in these societies is determined by complex, long-term social relationships that must be maintained, suggesting effective communication would be adaptive [22].

Horses are predominantly visual animals, with reasonable visual acuity that, at 23 cycles per degree, is better than domestic cats and dogs [23–25]. While horses’ use of head and body posture in signaling has been described in observational literature (e.g. [20] and [26]), surprisingly their use of facial expressions has been largely overlooked. This is despite attempts to quantify facial expressions in horses’ close relatives, plains zebra (*Equus quagga* [27]), and reports that horses do routinely use some apparently complex facial expressions (e.g. snapping and the estrous face, which both involve pulling back the lips and flattening of the ears [28]).

A systematic way of recording facial expressions would have a wide range of uses, with the potential to assess and improve welfare for horses, as well as enhancing our understanding of communication and cognition in this highly social species and providing insights into the effects of domestication. Questions about whether particular facial movements are associated with negative emotional states or indicative of positive experiences will be particularly important to address, and FACS provides an ideal framework for such investigations. More generally, FACS provides a framework through which species with different phylogenetic and ecological influences can be compared to investigate the functional significance and evolutionary origins of facial expressions.

### How Was FACS Modified for Use in Horses?

To modify FACS for use in non-human animals, the first step was to document and compare their facial anatomy [9–13, 29]. Horses are only distantly related to primates and the other animals FACS have previously been developed for; additionally horses have a dramatically different facial morphology to these animals, including laterally placed eyes, elongated faces, and raised zygomatic arches. Consequently, it is possible that the facial muscles of horses would show little similarity with the facial muscles of humans or those underpinning other FACS.

To facilitate valid comparisons across taxa, it is necessary to report findings in a consistent way, with a standardised nomenclature for the facial muscles [4]. While the anatomy of domestic mammals is generally well documented, it has been difficult to make direct comparisons between domestic animals and primates because of the inconsistent terminology used (see [4, 30] for reviews and a call for a standardized nomenclature for the facial muscles). Additionally, the anatomy of domestic mammals tends to be described from a veterinary perspective, so that the muscles of facial expression are often not documented comprehensively within one text (e.g. [31]). Therefore, we supplemented the previous literature on the facial anatomy of the horse with our own dissection using an innovative ‘face mask’ method, which allows a more complete view of the facial muscles and their attachments than traditional dissection methods [32–35]. In doing this we documented the muscles using the recommended terms, which allowed us to make direct comparisons between horses and the other species. The facial muscles of the horse are reported here using the standardised terminology [4], so that a free, detailed record of the muscles of facial expression is available alongside the EquiFACS manual.

We then analysed footage of a wide range of naturally occurring horse behaviours captured on high quality video. Discrete facial movements were identified, their proposed muscular basis was noted, and they were given a code in correspondence with previous FACS systems. All the facial movements identified are listed in the results, accompanied by a description of the appearance changes and video illustrations. Where necessary, the proposed muscular basis of the actions is given and there are also sections on subtle differences between similar actions.

## Method

### Anatomical Investigation of the Underlying Anatomy

The head of one horse was acquired from the New Bolton Centre at the University of Pennsylvania. The dissection was done using an innovative ‘face mask’ method, where the facial musculature is removed from the deep muscles and the skull with the skin, creating a ‘facial mask’ that holds all of the facial muscles (see S1 Text for full details of the dissection procedure). Using this novel approach preserves the superficially located facial muscles that might be lost in the traditional dissection method of removing the skin from the facial musculature. Furthermore, it provides a more complete picture of muscle attachments, by keeping superficial portions attached to the skin and deeper portions attached to the skull (see [32–35]). The muscles were examined for presence/absence, attachments to the bone, skin, and cartilage, as well as their three-dimensional relationships to one another and to the skull. Muscles were classified with reference to a variety of sources (mainly [36, 37]) and in relation to previous dissections completed for the development of FACS in other species [32–35]. All muscles and their attachments were recorded, sketched and photographed.

### Classifying Facial Actions

We collected and analysed 15 hours of video footage of a wide range of naturally occurring horse behaviours from a sample of 86 horses. Known ages ranged from 4 weeks to 27 years, and the sample included horses of different breeds, coat colours, and sex. Horses were videoed using a Canon XM2 video recorder in a variety of situations, including interacting with conspecifics, humans and other animals (e.g. dogs), feeding and mating.

Each discrete facial movement identified was given a code (an Action unit—AU—or an Action Descriptor—AD) in correspondence with previous FACS systems. Where EquiFACS identified movements analogous to those in humans or other animals, the same codes have been used. In some cases, although the same muscle is used in both humans and horses the resulting action on the face is very different; in these cases the Action Unit is prefixed with an ‘H’ e.g. AUH13. In other cases, although the action on the face is similar in horses and humans, the underlying muscular basis differs; in these cases the AU is prefixed by a 1 (e.g. AU122). Where novel movements were noted a new Action Unit or Descriptor was created (see Table 1 and Table 2). Agreement on these classifications was reached between two trained human FACS coders (JW and BMW), a comparative anatomist (AMB), and a specialist in animal behaviour (including horses) (KM).

**Table 1:** Summary of actions units in EquiFACS compared to Human FACS.

Action Unit	Muscles	In Human FACS
101 Inner brow raiser	Levator anguli occuli medialis	Resembles AU1, which is underpinned by the frontalis. The frontalis is present in horses (interscutularis) but does not seem to raise the brow region.
143 Eye closure and 145 Blink	Orbicularis occuli, Levator palpebrae superioris	Resembles AU43 and 45, but these are underpinned by the levator palpebrae superioris alone.
47 Half blink	Orbicularis occuli	Not described
5 Upper lid raiser	Levator palpebrae superioris	Same code and muscle
10 Upper lip raiser	Levator labii superioris, transverse nasi	Same code and muscles
12 Lip corner puller	Zygomatic major	Same code and muscle
113 Sharp lip puller	Levator labii superioris alaeque nasi	Not described, but similar visual properties to AU13.
H13 Nostril lift	Levator annuli oris fascialis	Same muscles as AU13, although different action and visual appearance
16 Lower lip depressor	Depressor labii inferioris	Same code and muscle
17 Chin raiser	Mentalis	Same code and muscle
18 Lip pucker	Orbicularis oris, incisvii labii	Same code and muscles, although more likely to occur in both lips in humans
122 Upper lip curl	Levator labii superioris; transverse nasi	Similar action to AU22, but more likely to occur in both lips in humans, and has a different muscular basis.
24 Lip presser	Orbicularis oris	Same code and muscle
25 Lips part	Depressor labii, or relaxation of the mentalis or orbicularis oris	Same code and muscles
26 Jaw drop	Masetter, temporal and internal pterygoid relaxed	Same code and muscles
27 Mouth Stretch	Pterygoids, digastric	Same code and muscles

**Table 2:** Summary of actions descriptors in EquiFACS compared to Human FACS.

Action Descriptor	In Human FACS
1 Eye white increase	Not described
101 Ears forward	Not described
102 Ear adductor	Not described
103 Ear flattener	Not described
104 Ear rotator	Not described
160 Lower lip relax	Not described
19 Tongue show	Same code
29 Jaw thrust	Same code
30 Jaw sideways	Same code
133 Blow	Similar to AD33, blow
38 Nostril dilator	Same code

The reliability of others to be able to learn EquiFACS and consistently code behaviour was tested with four coders, three of whom had no previous experience of FACS and one of whom had no previous experience of horses. The coders were each given the text, video examples, and practical instructions describing how to use EquiFACS effectively (see S3 Text) and asked to learn EquiFACS independently. The coders had minimum contact with the authors in this time, so that their performance would be considered a fair test of whether EquiFACS produces reliable coding when learned without training from the developers. When each coder was happy that they understood the principles of EquiFACS and felt they could reliably identify the expressions, they were asked to code 22 short video clips (containing the full range of AUs and EADs) with the manual for reference.

In accordance with previous FACS, reliability was measured using Wexler’s ratio [1, 8–12, 38]. Wexler’s ratio is a ratio of the number of agreements (i.e. the number of AUs on which coder 1 and coder 2 agreed x 2) divided by the number of possibilities for agreement (i.e. the total number of AUs scored by both coders). This is particularly suitable for situations, such as in FACS, where there are a large number of potential codes and there is likely to be a different number of items coded by each coder. Each person’s coding was compared to JW’s coding, the agreement ratio was calculated for each video clip, and then an average over the video clips was taken for each coder. The overall average was 0.86 (coder 1 = 0.85; coder 2 = 0.86; coder 3 = 0.87; coder 4 = 0.85), demonstrating high reliability across the coders.

### Ethical Statement

This study gained ethical approval from the University of Sussex Ethical Review Committee, and was carried out in accordance with the Association of Animal Behaviour Guidelines for the Treatment of Animals in Behavioural Research. All owners gave consent for their horses to be video recorded. It should be noted that the specimen for the dissection was not euthanised for the purpose of this study, but was acquired from a veterinary centre where the horse had been euthanised for clinical reasons.

## Results

### Facial Muscles of the Horse

**Fig 1:**
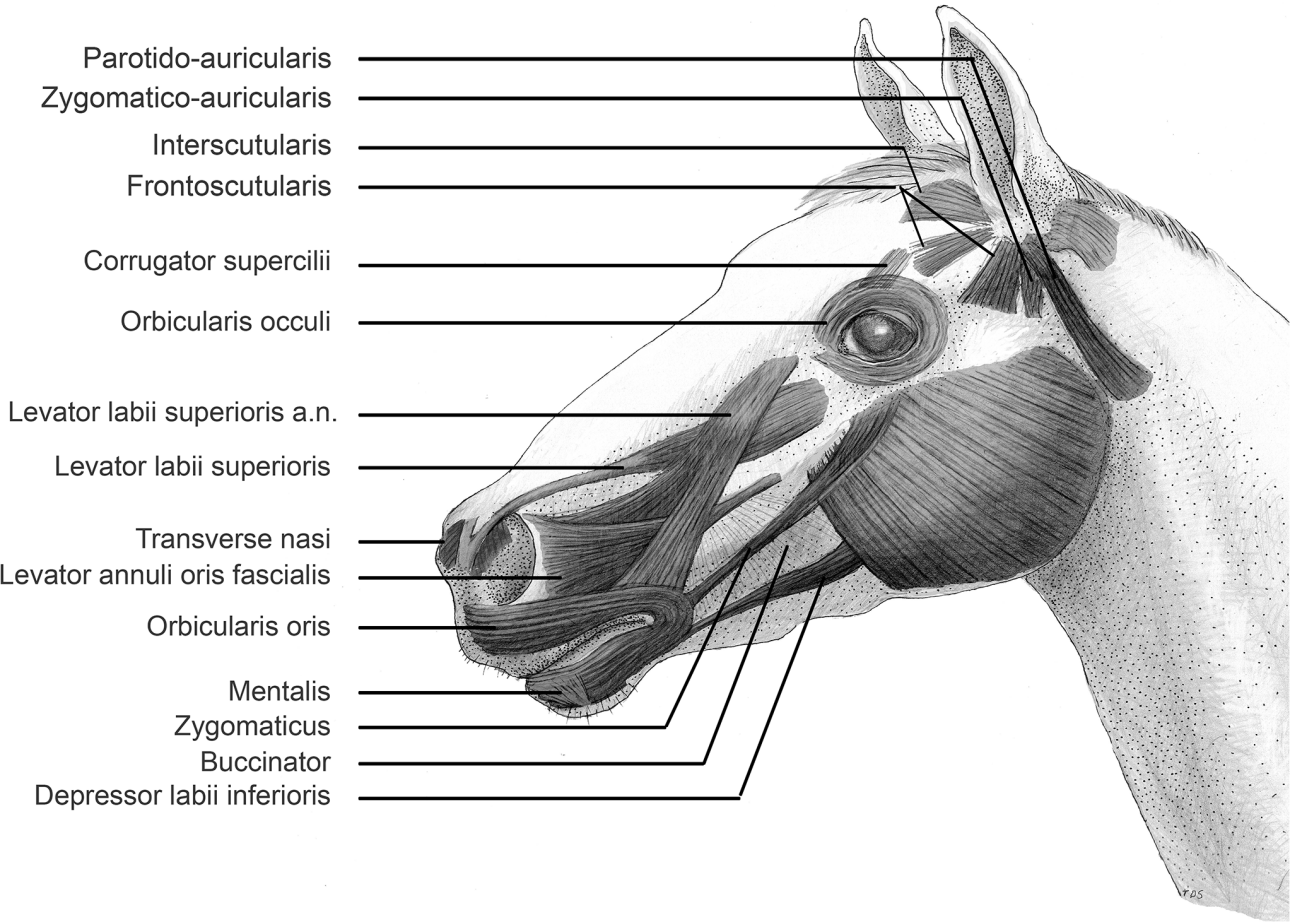
The facial muscles of the horse. NB. Levator labii superioris a.n. represents the levator labii superioris alaeque nasi, which is often also called the levator nasolabialis. Synonyms for the levator annuli oris fascialis muscle include the dilator nares muscle and the caninus muscle. The frontoscutularis has a frontal and a temporal arm.

Fig 1 depicts the facial muscles of the horse (S1, S2, and S3 Figs show the facial muscles located in our dissection). Overall, the facial muscles of horses are very complex, and although there are differences, there are a surprising number of similarities with humans and other primates. Table 1 summarises the findings in comparison to the human facial muscles. Detailed descriptions of the muscles are given in S2 Text.

Gross observations showed that the muscles around the ear, lips, and nose of the horse were particularly large and complex, with these muscles having many attachments to the fascia, cartilage, and other muscles (S2 and S3 Figs). In particular, many muscles of the lower face converged in the modiolus to form a large, complex muscular mass (S2 Figs). This was in contrast to previous suggestions that mammals distantly related to primates, such as Perissodactyla, should have very simple facial musculature, particularly around the lips and mid-face [39, 40]. The orbital and mid-face region of the horse had numerous muscles including both a zygomatic major and minor (also termed the malaris), and an extensive obicularis occuli, although the muscles in these areas were thinner than the muscles around the ears and lower face.

There was a large amount of adipose compared to non-human primates, e.g. chimpanzee, [33]; rhesus macaques, [34] Otolemur [32] and Hylobatids [35], possibly due to the domestic situation of the horse. The adipose was particularly prevalent around the ear, but also found around the mouth (this has been cut away in the Figs to allow better observation of the muscles).

## Facial Actions of the Horse

### Upper Face Action Units

#### Action Unit 101: Inner Brow Raiser

**Fig 2:**
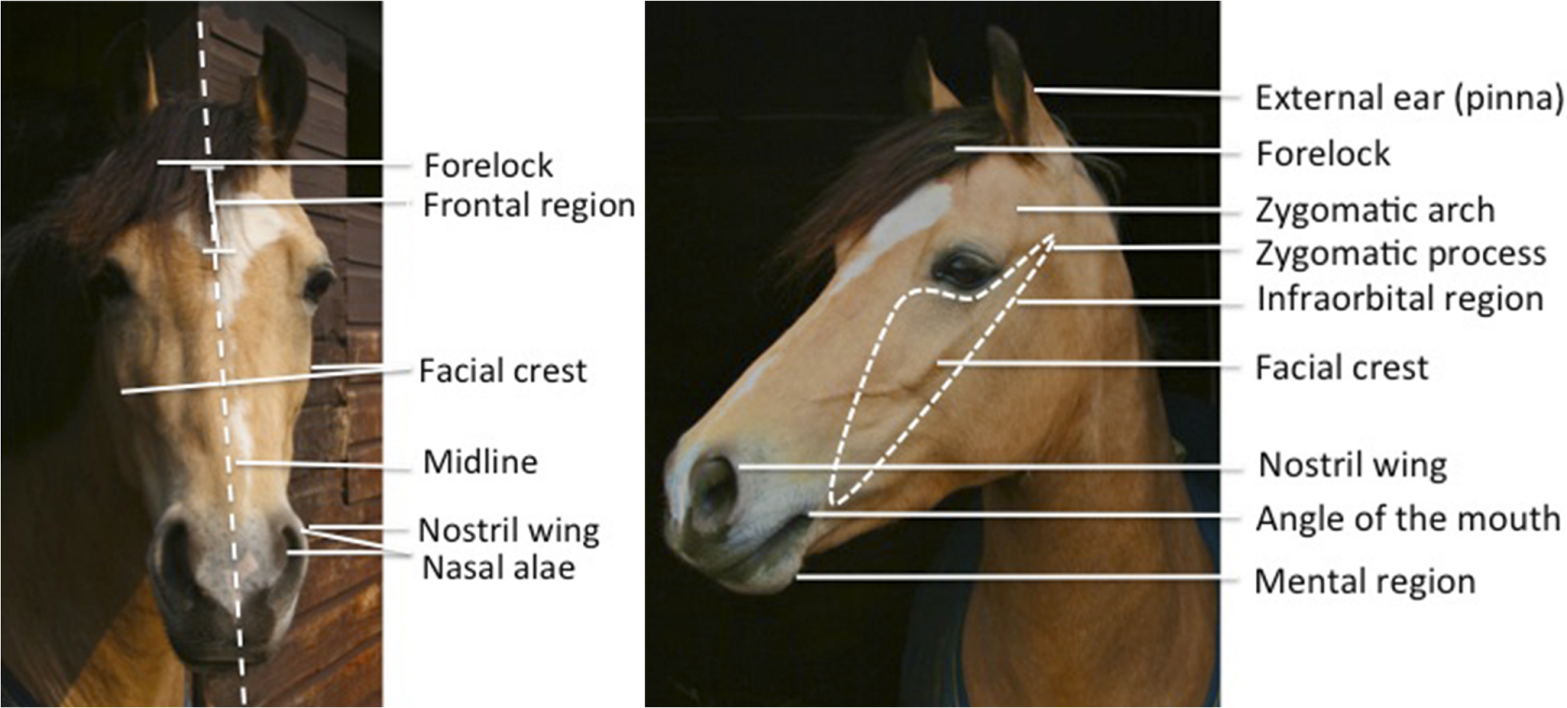
The facial landmarks of the horse.

**Fig 3:**
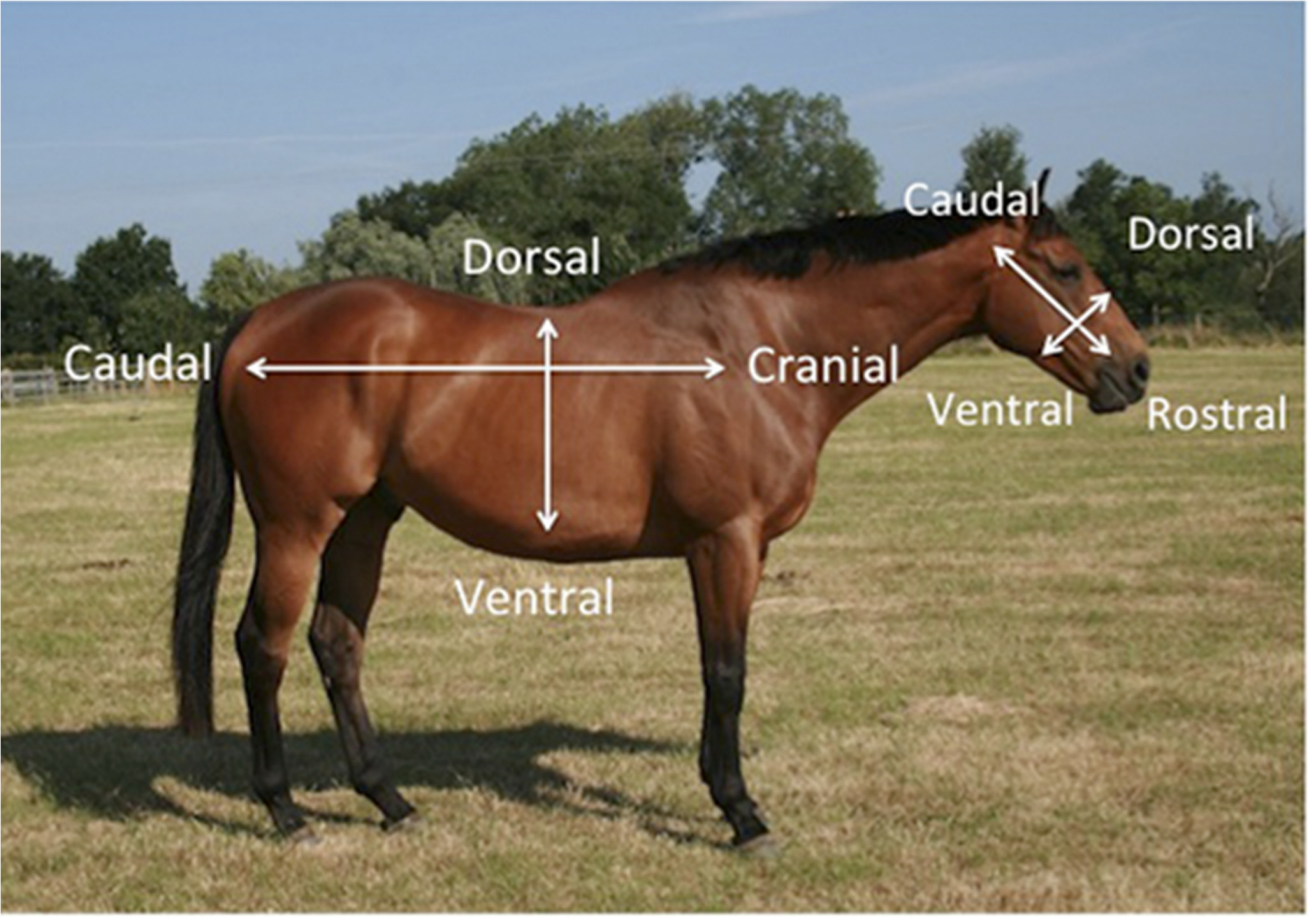
A guide to anatomical direction.

The anatomy of the eye area is different in horses as compared to humans, other primates, and dogs (see Fig 2 for a visual representation of the key facial landmarks of the horse and Fig 3 for a guide to anatomical direction). Horses do not have the features, such as eyebrows or the prominent brow ridge, that are thought to accentuate brow movements in other animals. However, horses do have a facial action that raises skin above the inner corner of the eye. This is similar to the movement derived from the frontalis in humans (inner brow raiser—AU1), although in horses the action is underpinned by the levator anguli occuli medialis and the corrugator supercilii, making it analogous to the inner brow raiser seen in dogs and cats. Consequently, the code used to denote this movement in dogFACS and catFACS (AU101) is also used here.

**Fig 4:**
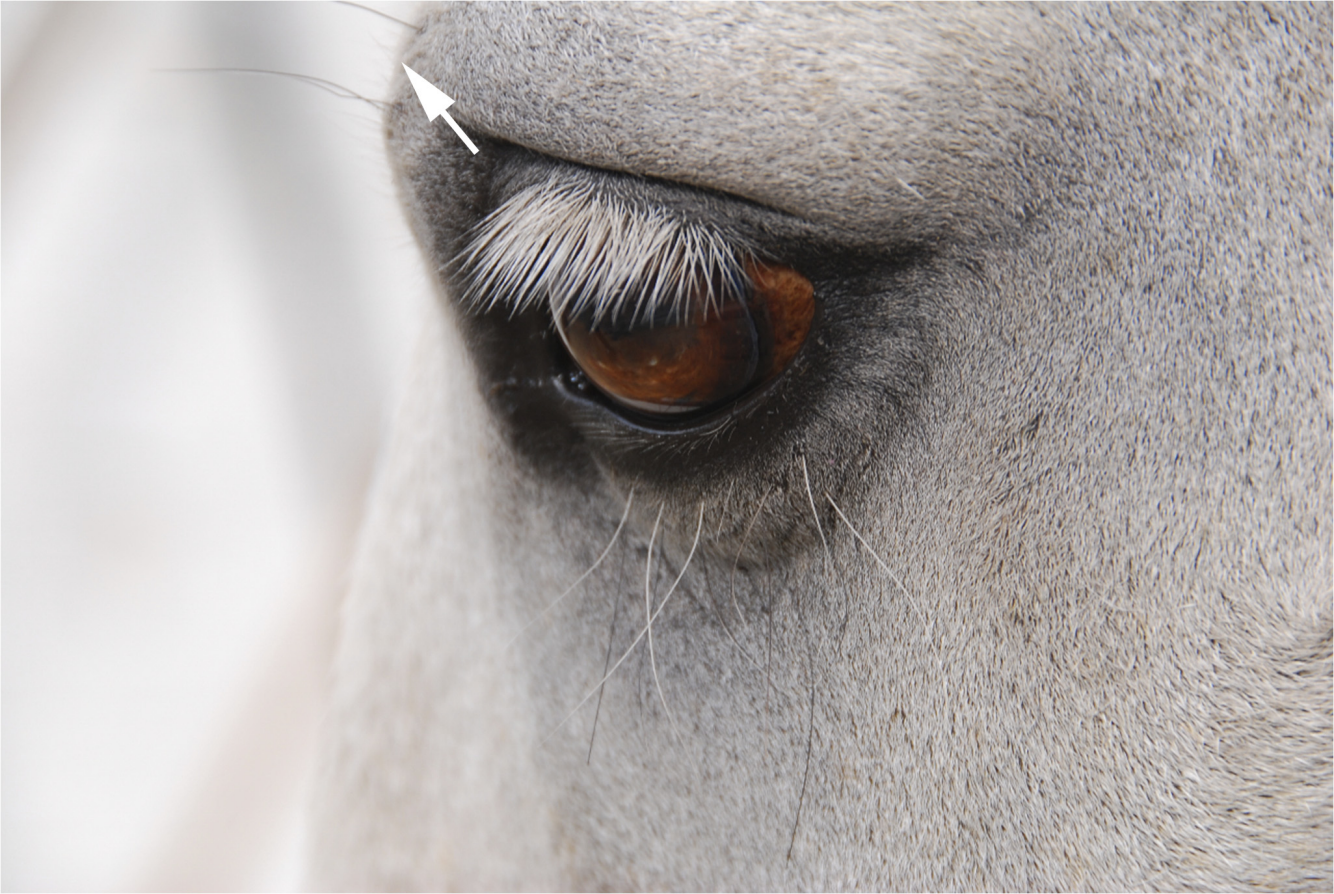
The area and direction of movement in AU101, inner brow raiser. The picture shows the left eye of the horse.

**A. Proposed muscular basis:** levator anguli occuli medialis muscle, corrugator supercilii muscle (Fig 1, see S2 Text for additional comments on these muscles).
**B. Appearance changes:**
  1. The skin above the inner corner of the eye is pulled dorsally and obliquely towards the medial frontal region (see Fig 4).

2. The skin may bulge above the inner corner of the eye.
3. Wrinkles can appear (or if already present deepen) in the area above the inner eye.
4. The shape of the skin above the eye changes: instead of following the shape of the eyelid an angular contour develops.
5. It may accompany an eye movement, although does not necessarily.
6. This action is quite subtle and can be difficult to reliably discern from a distance.
**C. Minimum criteria to code AU101:** dorsal movement of the skin above the inner eye region. See S1 and S2 Videos for examples of AU101.

#### Action Units 143, 145, and 47: Eye Closure, Blink and Half Blink

Horses have three eyelids: upper, lower, and third (nictitating membrane). However, the third eyelid is usually only barely visible at the inner corner of the eye. The eyes of the horse are placed more laterally than the eyes of primates and dogs (40 degrees) [41], with no prominent supercilliary arch or epicanthal fold.

In horses, the orbicularis occuli muscle is mostly responsible for the actions of closing the eyes and blinking. This is in contrast to humans and other primates, who can close the eyes by relaxation of the levator palpebrae superioris alone. The eye closing action seen in horses more resembles that seen in dogs and cats, and the AUs given below correspond with the parallel actions in dogFACS and catFACS.

AUs 143 and 145 both refer to closure of the eyes, although they are mutually exclusive and cannot be coded simultaneously. The main difference between these actions is the speed at which they occur, and the extent of recruitment of the orbicularis oculi. AU143 is substantially slower than AU145. Additionally, AU145 is seen in conjunction with higher degrees of actions that resemble AU6 (the cheek raiser) in humans (e.g. raising of the infraorbital region).

However, AU6 and AU7 (lid tightener) are both omitted from EquiFACS. Both of these movements constrict the skin around the eye in primates, but are not reliably discernable as independent facial movements in horses. Horses do show some signs of AU6 within AU143 and 145 (contraction of the infraorbital region) and some contraction of the orbicularis occuli is seen that reduces the opening of the eye without closing it completely. However, these contractions are not reliably discernable as independent actions, and would be very difficult to distinguish, particularly in the field. For this reason, AU47 – the half blink, has been created, which covers all instances where the eye partially closes.

EquiFACS omits AU46, which represents a wink in humans, as AU46 refers to an intentional action. The absence of AU46 from EquiFACS is to avoid subjective decisions about whether there is intentionality of expression in horses.

#### Action Unit 143: Eye Closure

AU 143 denotes eye closure that lasts for more than half a second. This movement results from the relaxation of the levator palpebrae superioris muscle, and small actions of the orbicularis oculi.

**A. Proposed muscular basis:** contraction of the orbicularis oculi muscle and relaxation of the levator palpebrae superioris muscle (Fig 1, see S2 Text for additional comments on these muscles).
**B. Appearance changes:**
  1. The upper and lower eyelids move towards each other to close the eye.
  2. More surface of the upper eyelid is exposed than usual.
  3. When the eyelids meet and the eye is closed, some tension might be exhibited in the skin covering and surrounding the eye.
  4. The skin located above and particularly below the eye may be pulled inwards. This is most obviously seen in the infra-orbital area, which contracts superiorly (both medially and laterally).
  5. Once closed the eyelids may flutter slightly (e.g. if falling asleep) and the code AU143 covers these slight movements. However, if the eyes then open fully, code subsequent movements as new movements.
**C. Minimum criteria to code AU143:** the eyelids must be drawn together to close the eye, and the eye must remain closed for more than half a second. See S3 and S4 Videos for examples of this action.
**D. Subtle differences between AUs:** AU143 is slower than AU145 (below), and the eyes close for more than half a second. If the eyes close for less than half a second score AU145. If the eyes do not close completely then do not score AU143, instead score AU47.

#### Action Unit 145: Blink

AU145 refers to a quick eye closure that involves both the relaxation of the upper eyelid and some contraction of the lid tightening muscles. Both the timing of the action and the appearance of the eyelids, particularly the lower eyelid, can differentiate it from AU143.

**A. Proposed muscular basis**: contraction of the orbicularis occuli muscle and relaxation of the levator palpebrae superioris muscle (Fig 1, see S2 Text for additional comments on these muscles).
**B. Appearance changes:**
  1. The upper and lower eyelids move towards each other to close the eye.
  2. There is a rapid sequence of actions in which the upper eyelid relaxes and the lid tightening muscles contract to close the eye, followed immediately by the reversal of these actions to open the eye. There is no pause or hesitation when the eyes are closed.
  3. When the eyelids meet and the eye is closed, some tension might be exhibited in the skin covering and surrounding the eye.
  4. The skin located above and particularly below the eye may be pulled inwards. This is most obviously seen in the infra-orbital area, which contracts superiorly (both medially and laterally).
**C. Minimum criteria to code AU145:** both eyelids must move together to cover the eye, and this action must be reversed within half a second. See S4 and S5 Videos for examples of this action.
**D. Subtle differences:** AU145 is faster than AU143—the eyes close for less than half a second and there is no pause while they are closed. If the eyes do not close completely then do not score AU145, instead score AU47 (half blink).

#### Action Unit 47: Half Blink

This movement has not been observed in humans, so there is no corresponding AU. Interestingly there is a corresponding movement in cats however, and the same code, AU47, is also used here.

**A. Proposed muscular basis:** orbicularis oculi muscle (Fig 1, see S2 Text for additional comments on these muscles).
**B. Appearance changes:**
  1. Reduction of the eye opening by the eyelids drawing the eyelids or the skin around the eye contracting.
  2. Although the opening of the eye is reduced, the eye does not close completely.
  3. The skin located above and particularly below the eye may be pulled inwards. This is most obviously seen in the infra-orbital area, which contracts superiorly (both medially and laterally).
**C. Minimum criteria to code AU47:** a reduction in the opening of the eye. See S6 and S7 Videos for examples of this action.
**D. Subtle differences:** this AU differs from AU143 and AU145 because the eye never closes completely. Be cautious that the eye opening is reduced due to the movement of the eyelids towards each other, and the action is not just release of AU101, inner brow raiser (see S7 Video).

#### Action Unit 5: Upper Lid Raiser

AU5 pulls the upper eyelid back into the eye socket. In the usual eyes open position, the upper eyelid covers the eye to some extent and there is some contraction of the muscle that underlies AU5. However, AU5 denotes when the contraction goes beyond the usual, pulling the eyelid further back into the eye socket. In humans this action is very obvious, however in horses there is less of a prominent brow ridge and this action is more difficult to discern. The position of the eyelashes can be very helpful in determining the position of the eyelid.

**A. Proposed muscular basis:** levator palpebrae superioris muscle (Fig 1, see S2 Text for additional comments on this muscle).
**B. Appearance changes:**
  1. Widens the eye opening.
  2. Raises the upper eyelid so that it is pulled caudally and dorsally.
  3. The amount of the upper portion of the eyeball exposed increases. Sclera above and around the iris may also be exposed (or increased is already present in a neutral position).
  4. The shape of the upper eyelid changes as portions medially and/or laterally are pulled up.
**C. Minimum criteria to code AU5:** an increase in the eye opening caused by the raising of the upper eyelid. See S8 and S9 Videos for examples of this action.

#### Action Descriptor 1: Eye White Increase

Horses do have white sclera around the eyes, although generally this is not visible at rest. In some situations, horses will display more of the white sclera due to a change in the opening of the eye or position of the eyeball. As this is not underpinned by a particular muscular basis, it has been labeled as an Action Descriptor, AD1. This is coded separate to AU5, as although there may be an increase in visible sclera with AU5 it is not a requirement for coding AU5. Additionally, AU5 represents widening eye opening, whereas movements of the eyeball unrelated to AU5 can also increase the visible white sclera. Consequently, AD1 and AU5 can both be scored independently, but also simultaneously.

The amount of visible white sclera is associated with the expression of fear in many animals, including humans[42, 43]. Consequently, AD1 is an essential part of EquiFACS. However, depending on the degree of interest there are two levels of coding for this action. One code simply represents an increase in the amount of white sclera exposed. The second method (which is optional and may only be useful if there is a specific interest in this expression) allows a calculation of the percentage of eye-white visible (see S4 Text).

It is necessary to establish a suitable baseline for coding these movements, as different horses will display varying amounts of white sclera at rest. It is also important to be wary of coding these movements alongside movements of the head or camera viewpoint, as these factors can also influence the amount of sclera visible

**B. Appearance changes:**
  1. The white sclera becomes visible, or if present at rest there is an increase in the amount noticeable.
  2. The white sclera may increase in any part of the eye. It is most often seen on the medial dorsal edge, but can increase around the whole circumference of the iris.
  3. The eye opening may widen.
  4. The eyeball may move in the socket.
  5. In some horses the third eyelid (nictitating membrane) can initially appear to be white sclera in the rostral and ventral corner of the eye. If possible, obtaining a close up view should help to establish what is sclera and what is nictitating membrane.
**C. Minimum criteria to code AD1:** an increase in the percentage of white sclera visible. See S8, S9, and S10 Videos for examples of this action.
**D. Subtle differences:** the critical component of AU5 is the increased opening of the eye. Although this may reveal more visible eye white, increased white sclera can also be seen without the eye widening (in this case, code only AD1).

### Lower Face Action Units

The horse has an elaborate network of robust facial muscles in the lower face, with a complex innervation. This has some similarities with the facial musculature of humans, however horses also have a number of different specialisations (see Table 1). The movements of the lower face are complicated and often many actions are seen together in a rapid, fluid sequence.

#### Action Unit 10: Upper Lip Raiser

**Fig 5:**
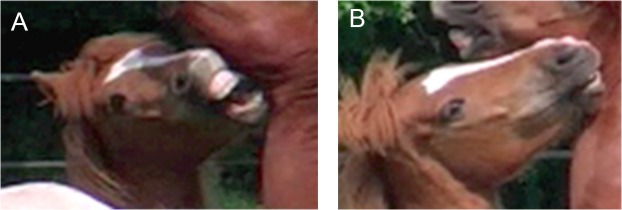
AU10, upper lip raiser (+ 16 + 17 + 25 + 27 + AD1 [AD1 seen in part B only]). Part A shows appearance changes 1, 2, 3, 4, and 7; part B shows appearance changes 2, 4, 5, 6, and 7.

**A. Proposed muscular basis:** levator labii superioris alaeque nasi muscle (also called levator nasolabialis), transverse nasi muscle (Fig 1, see S2 Text for additional comments on these muscles).
**B. Appearance changes:**
  1. The centre of the upper lip is raised straight up, and in strong actions the rest of the upper lip may also be partially raised (see Fig 5).

2. The upper teeth, and in strong actions the upper gum, will become visible.
3. The upper lip does not evert as it does in the lip curl (AU 122) or protrude away from the lip as in the lip pucker AU18, (unless AU10 is combined with these movements). Instead, the upper lip edge may appear to roll inwards.
4. Transverse wrinkles are seen across the front of the nose, and these may extend up the face (except in very mild actions). These wrinkles are most obvious from a frontal view, but in strong actions the corrugation of the skin can also be seen in profile.
5. Wrinkles may also appear behind the caudal edge of the nostril wing.
6. Although the wrinkles may extend up the nose, and there may be some tension in the skin of the midface, AU10 does not affect the infraorbital region or the skin around the eyes.
7. The nostril wings may widen and rise. However, this is only due to the movement of the upper lip displacing the skin in the nostril area, rather than any movement of the nostril wing itself.
**C. Minimum criteria to code AU10:** the central part of the upper lip is raised. See Fig 5 and S11 and S12 Videos for examples of this action.
**D. Subtle differences:** similar transverse wrinkles occur across the bridge of the nose in both AU10 and AU122, upper lip curl. Additionally, both actions raise the upper lip exposing the teeth and gums. However, the lip does not evert in AU10, whereas the lip curls up and round in AU122. If the inside of the upper lip (rather than just the gum) is visible, code AU122 instead of AU10. Movement of the nostril wings with AU10 are in conjunction with the movement of the upper lip. This is unlike the movement of the nostril seen in AUH13, nostril lift, which is isolated to the nostril wing. It is difficult to code AU10 and AUH13 together unless a very strong action of AUH13 has occurred or the actions occur sequentially.

#### Action Unit 12: Lip Corner Puller

In primates, dogs, and cats, the zygomatic major muscle (often termed zygomaticus muscle in dogs and cats) muscle pulls the lip corners obliquely towards the cheekbone. Horses also have a zygomatic major muscle (generally termed the zygomaticus muscle) that pulls the lip corners back. However, this action has a slightly different appearance in horses due to their different facial morphology.

**A. Proposed muscular basis:** zygomatic major muscle (also termed zygomaticus muscle—Fig 1, see S2 Text for additional comments on this muscle).
**B. Appearance changes:**
  1. The lip corners are pulled back caudally.
  2. This may expose the teeth and gums.
  3. In strong actions, the medial section of the lips may also be drawn back exposing the teeth and gums. However, this is due to the stretching of the skin of the lips, rather than a genuine movement up or down. Consequently, even when the middle of the lips are drawn back by AU12, wrinkles on the nose or chin boss are not seen. If these signs are evident consider coding AU10, upper lip raise, or AU16, lower lip depressor, in addition.
  4. The mouth is often open or repeatedly opening and closing with this movement.
  5. The lip corners may pouch and wrinkle.
  6. The lips are elongated.
  7. The action of AU12 is not seen on the rest of the face, as it is in primates. There is no raising of the infraorbital region.

**C. Minimum requirement to code AU12:** the corners of the lips must be pulled towards the ears. See S13, S14, and S15 Videos for examples of this action.
**D. Subtle differences:** AU12 is likely to be confused with (or act with) AU113 – see section on subtle differences in AU113.

#### Action Unit 113: Sharp Lip Puller

This action resembles AU13, sharp lip puller, in humans, where the corners of the lips are pulled sharply up towards the upper jawbone. However, in horses a different muscle to that used in humans causes this movement. To account for this differing muscular basis, this action has been given the code 113.

**Fig 6:**
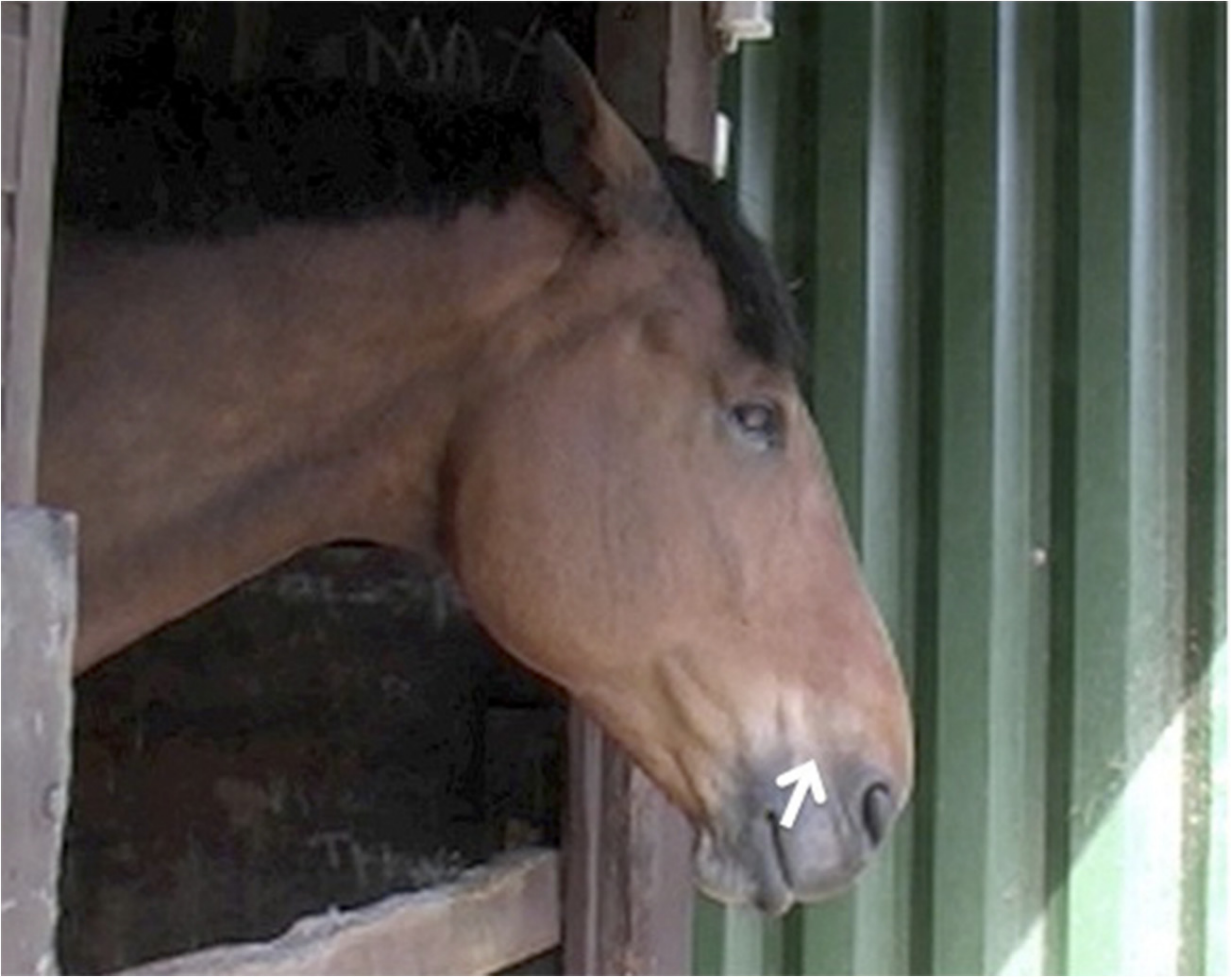
The location and direction of movement in AU113, sharp lip puller (indicated by the arrow). The lips are in the neutral position. AU113 is very difficult to code from photographs unless there is a very strong action, or it is combined with AU12, lip corner puller.

**A. Proposed muscular basis:** levator labii superioris alaeque nasi muscle (also known as the levator nasolabialis, see Fig 1 and S2 Text for additional comments on this muscle).
**B. Appearance changes:**
  1. The corner of the upper lip is pulled up towards the bridge of the nose (Fig 6).

**Fig 7:**
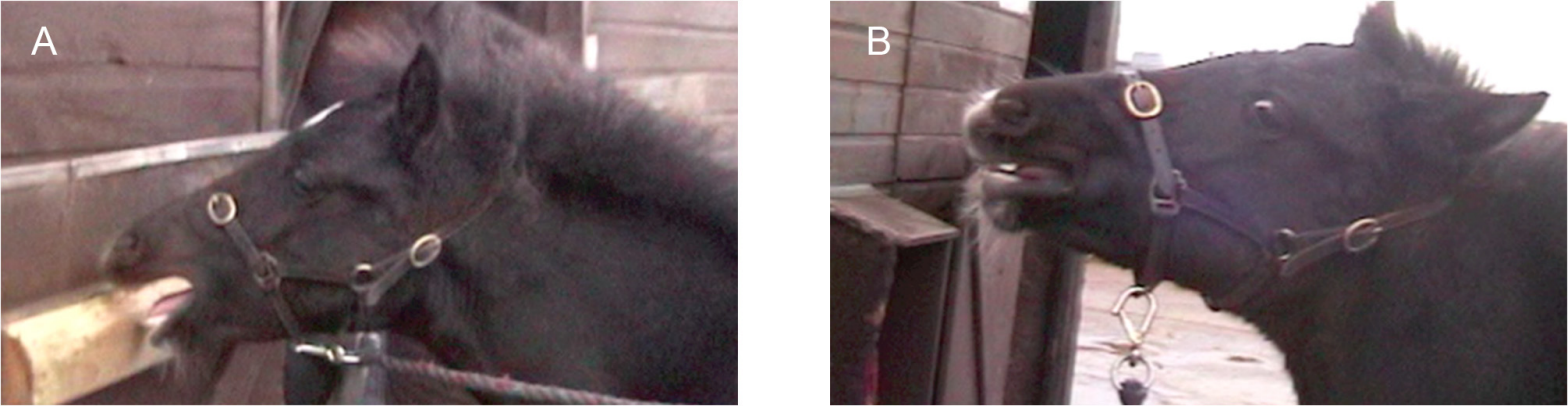
AU12, lip corner puller, without AU113, sharp lip puller, (A) and AU12 with AU113 (B). Note the difference in the shape at the corner of the mouth. AU12 produces a curvature at the mouth corner; however, when AU113 is applied with AU12 the mouth has an angular appearance at the top corner, with corresponding wrinkles in the skin surrounding the mouth. See S15 Video for a demonstration of these actions acting simultaneously.

2. The medial section of the upper lip does not rise (unlike AU10, upper lip raiser; see section on subtle differences below). This means that AU113 is only discernable from a profile view, as there is little or no observable action from a frontal perspective.
3. The skin above the upper lip, extending caudally up the face, also raises and wrinkles may develop in this area.
4. Wrinkles running along the face may appear under the nostril wing.
5. There may be some bulging under the skin in the area where the levator labii superioris alaeque nasi lies.
6. This can be a very discrete movement, although is generally more clear if the video clip is watched in slow motion.
7. Some tension may be exerted on the lower lip and the skin around the mouth. However, this tension is only minimal and in the direction of the upper lip movement. If there is movement of the lower lip in other directions, then consider coding additional AUs.
**C. Minimum criteria to code AU113:** oblique (caudal and dorsal) movement of the lateral part of the upper lip. See S14, S16, and S17 Videos for examples of this action.
**D. Subtle differences:** AU113 differs from AU12 in the direction of the movement. In AU12 the skin is drawn back at the corners of the mouth, whereas in AU113 the skin is drawn up towards the bridge of the nose. AU113 and AU12 do sometimes occur together, but when this happens the actions occur sequentially with AU113 generally occurring first, and so the timing of the actions can help distinguish them. Additionally, when AU113 is acting with AU12 the curve at the corner of the mouth becomes much more angular (Fig 7). AU113 also differs from AU10, because the movement is restricted to the corners of the lips so the medial section of the upper lip does not rise.

#### Action Unit H13: Nostril Lift

In humans the levator annuli oris fascialis muscle (also known as the caninus muscle or dilator naris lateralis muscle) acts on the upper lip and is responsible for AU13. However, in horses the levator annuli oris fascialis has no action on the upper lip. Instead, it draws back the caudal wing of the nostril. Consequently, although this movement has the same muscular basis in horses and humans, it is labeled AUH13 in EquiFACS to highlight the difference in action and appearance.

**Fig 8:**
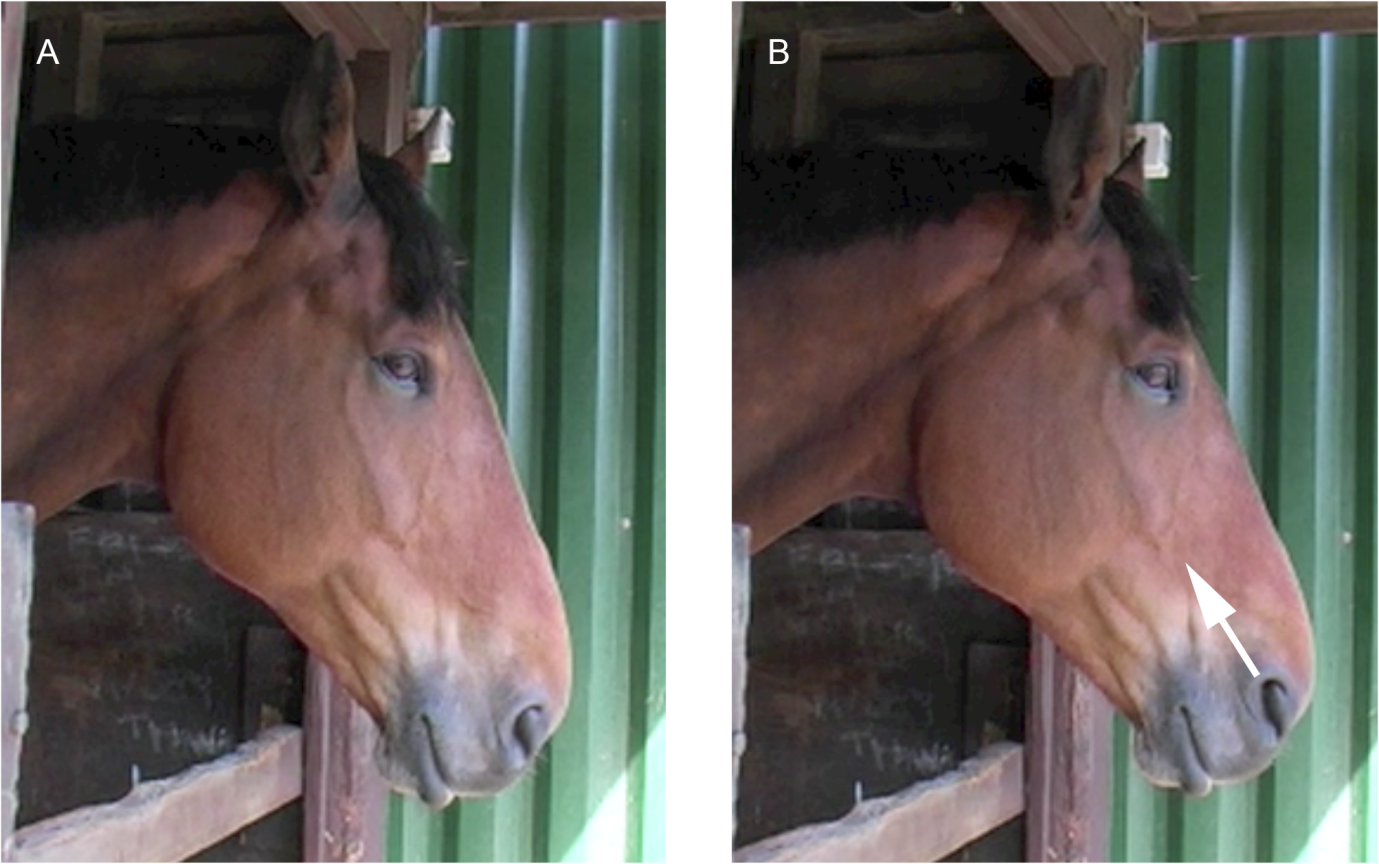
Direction and area of AUH13, nostril lift. The picture on the left shows the neutral face, the picture on the right shows the face with a mild action of AUH13 applied. The arrow illustrates the location and direction of movement.

**A. Proposed muscular basis:** levator annuli oris fascialis muscle (also known as the caninus muscle or dilator naris lateralis muscle, see Fig 1, see S2 Text for additional comments on this muscle).
**B. Appearance changes:**
  1. The caudal (back) edge of the nostril is pulled up and drawn round laterally (Fig 8).

2. This changes the shape of the nostril at this edge, from a smooth curve to a more angular shape.
3. The nostril is elongated.
4. AUH13 can cause the skin behind the nostril to wrinkle, or cause wrinkles to deepen if already present. This appearance change is particularly useful for determining weaker actions of AUH13.
5. Some horses may hold this movement on the face for a long time, and it might become confused with the neutral shape of the nostril. As there is no movement that can lower the caudal edge of the nostril, a drop in the nostril wing indicates that AUH13 was present.
6. This movement can be unilateral and often is seen performed on only one side of the face. For this reason, unless you can see both sides of the face and are certain that a bilateral movement has occurred code a unilateral action of AUH13.
7. As this movement produces a very distinctive shape to the nostril, it is possible to code this from photographs when it has been applied at high intensities.
**C. Minimum criteria to code AUH13:** the caudal edge of the nostril must move laterally and up, or if already present on the face the caudal edge of the nostril must drop down as the levator annuli oris fascialis muscle relaxes. See Fig 8 and S18 and S19 Videos for examples of this action.
**D. Subtle differences:** AUH13 is most likely to be confused with AD38, nostril dilator. One key difference is that AUH13 will not act on the rostral (front) edge of the nostril, whereas AD38 will. If there is some action on the rostral edge of the nostril, consider coding AD38 instead of (or, if sequential actions are seen, in addition to) AUH13. Additionally, in AUH13 there will be definite caudal movement of the skin behind the nostril wing, which is not seen in AD38.

#### Action Unit 16: Lower Lip Depressor

AU16 refers to the action of the lower lip being pulled down by the depressor labii inferioris muscle, as it does in primates. It must be visibly pulled down, rather than just relaxed. If the lower lip is hanging down due to relaxation, then code AD160, lower lip relax. The images, video, and descriptions deal with AUs 16+25, lips part. Usually, when there is an action of 16 the lips part and are scored as 16+25, 16+25+26, jaw drop, or 16+25+27, mouth stretch.

**A. Proposed muscular basis:** depressor labii inferioris muscle (Fig 1, see S2 Text for additional comments on this muscle).
**B. Appearance changes:**
  1. The lower lip is pulled down ventrally.
  2. The lower lip is stretched and pulled down laterally.
  3. The skin covering the mental region can be stretched laterally and down, flattening and occasionally wrinkling the skin in this area.
  4. In stronger movements this will expose some of the teeth and gums.
  5. The pink flesh on the inside of the lower lip may show.
  6. May cause the lower lip to protrude or flatten (depending on the individual).
  7. It is often seen in combination with other AUs, and when seen with AU17 the combination produces a distinct shape–see the sections on subtle differences.
  8. Often 16 may present more strongly on one side of the face than the other. However, only code a unilateral action if signs of 16 are completely absent from one side of the face.
**C. Minimum criteria to code AU16:** the lower lip must be pulled down. See S20 and S21 Videos for examples of this action.
**D. Subtle differences:** AU16 is a definite movement, whereas when the lip is lowered by AD160, lower lip relax, it is due to relaxation of the muscles. AU16 is often combined with AU17, chin raiser, which changes the appearance of the lower lip–see the details of combination AU16 + 17 and **S21 Video.** Finally, when the mouth is open or the skin around the mouth is stretched (e.g. in AU12, lip corner puller) then the skin of the lower lip will be stretched and some of the lower teeth or gum might show. To code 16 in these situations try and establish whether the lowering of the lip is greater than would be expected by skin stretching or mouth opening alone.

#### Action Descriptor 160: Lower Lip Relax

As in chimpanzees, this action occurs from the relaxation of the lower lip and the lip is pulled down by its weight alone. See S22 Video for an example of this action.

**Fig 9:**
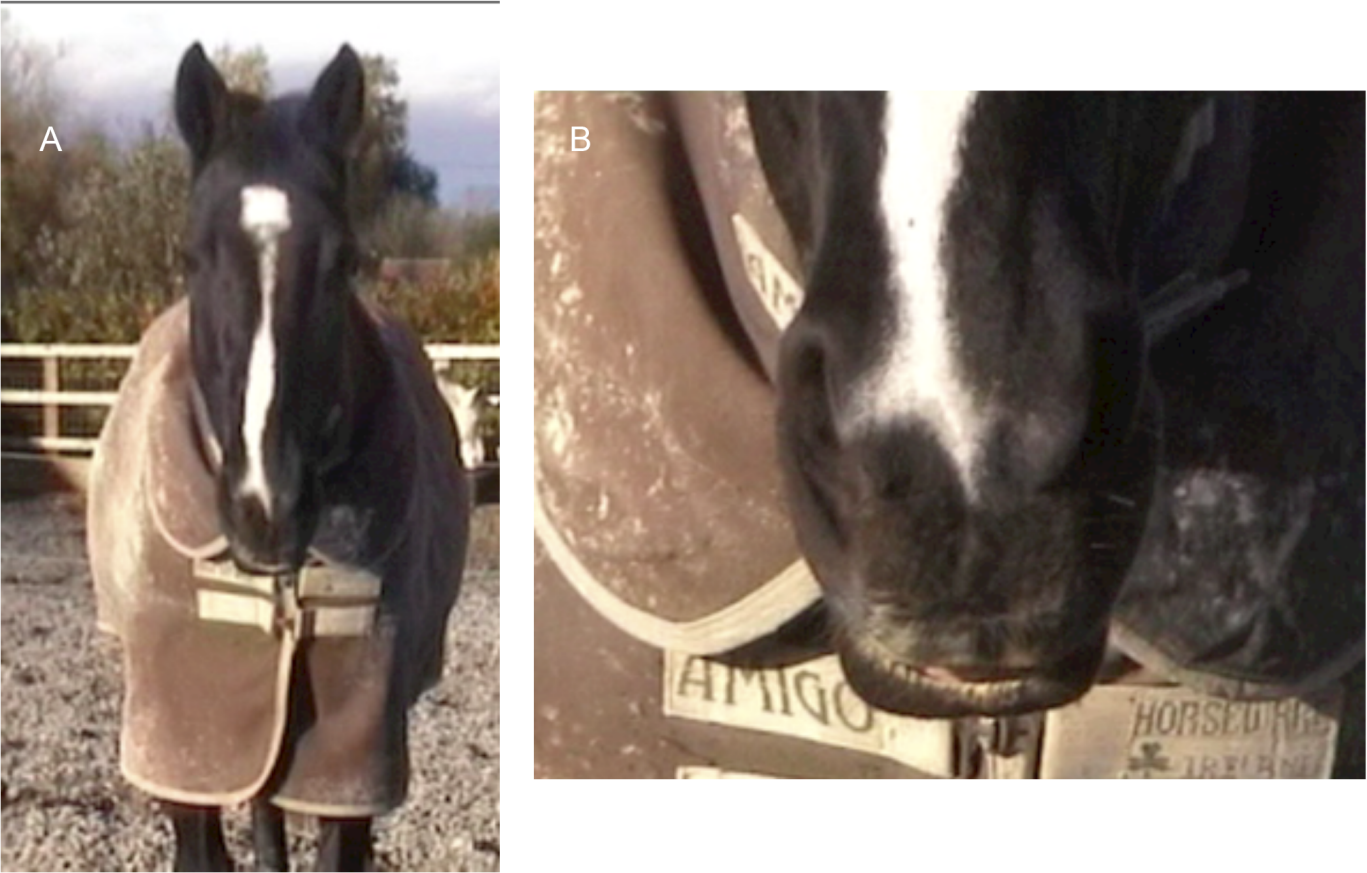
AD160, lower lip relax, seen from a distance and a close up view. Note that from a distance, although subtle, AD160 can be identified by the visible pink flesh from the inside of the lower lip.

**B. Appearance changes:**
  1. The lower lip is visibly relaxed and hangs loose with no tension.
  2. Often this will cause the lips to part and some of the teeth and gums may become visible.
  3. Similarly, the pink flesh on the inside of the lower lip may show (see Fig 9).

**Fig 10:**
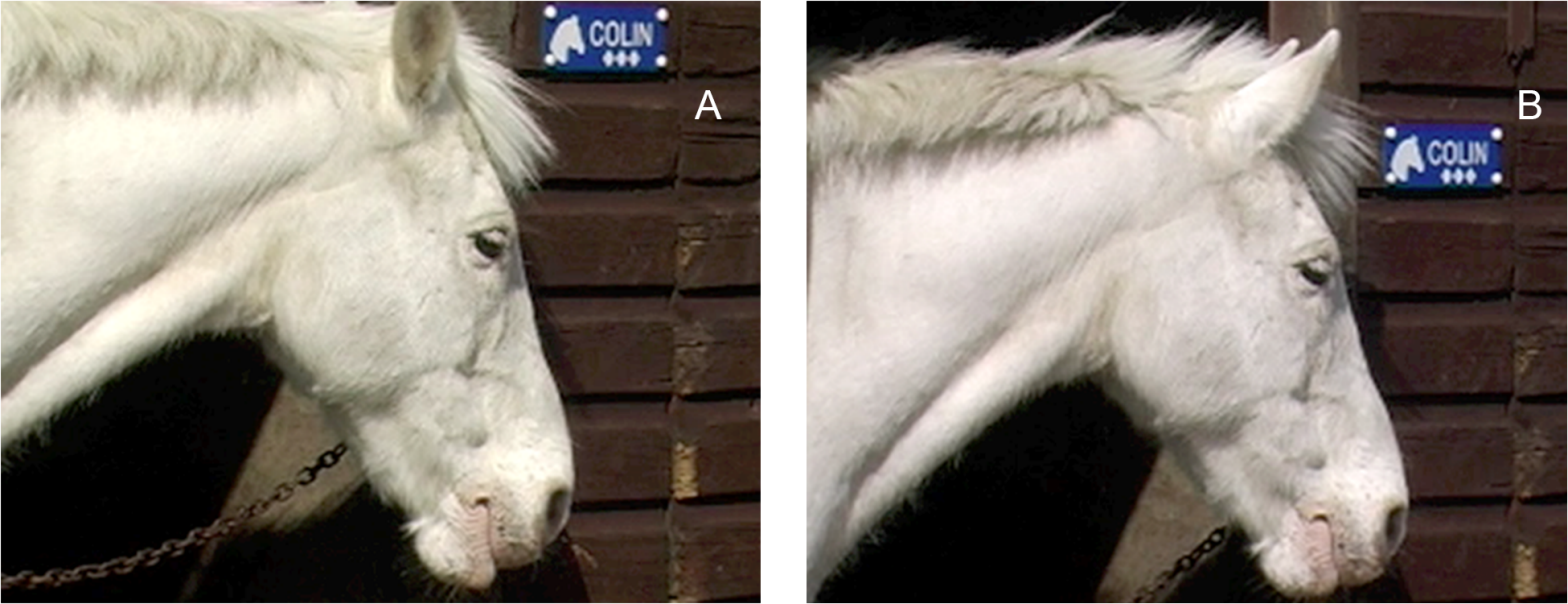
Profile view of AD160, lower lip relax. Panel A demonstrates the outline of the lower lip when AD160 is present, and panel B when AD160 is released.

4. The outline of the lower lip will change; there will be less definition in the lip and the lower lip will not meet the upper lip (see Fig 10). Establishing a neutral position for the lower lip can help in cases where AD160 is slight.

5. As this action is a relaxation of the face, rather than the application of a specific muscle, the onset of this action can be gradual. However, often the release of this action (when the lower lip tightens to resume the neutral position) is much more defined. Watching for the release of AD160 can help to establish whether it was present or not.

#### Action Unit 17: Chin Raiser

AU17 is underpinned by contraction of the mentalis muscle, which arises in the prominence of the mental region (also known as the chin) and inserts into the mental region. Consequently AU17 tightens the skin in the mental region and pushes the skin up towards the lower lip and nose.

**A. Proposed muscular basis:** mentalis muscle (Fig 1, see S2 Text for additional comments on this muscle).
**B. Appearance changes due to AU17:**
  1. The skin covering the mental region tightens and moves up towards the upper lip. This alters the outline of the lower lip, making the mental region more defined.
  2. The skin covering the mental region and lower lip are pushed upwards.
  3. Wrinkles may appear in the mental region as the skin is stretched and tightened.
  4. The lower lip may protrude, particularly in strong actions. This may cause a prominent ridge under the lower lip.
  5. This action of the lower lip may also push the upper lip up slightly.
**C: Minimum criteria to code AU17:** the skin covering the mental region and lower lip must be pushed up. See S23 and S24 Videos for examples of this action.
**D. Subtle differences:** see sections following AU16 and combination AU16 + 17.

#### Action Unit Combination 16 and 17

In combination AU16 + 17 the action of the depressor labii inferioris muscle (pulling down the lip) is mediated by the action of the mentalis muscle (pushing up the skin of the mental region). This causes a distinct appearance, where the lip can still be pulled down, but to a lesser extent than when AU16 is acting alone.

**Fig 11:**
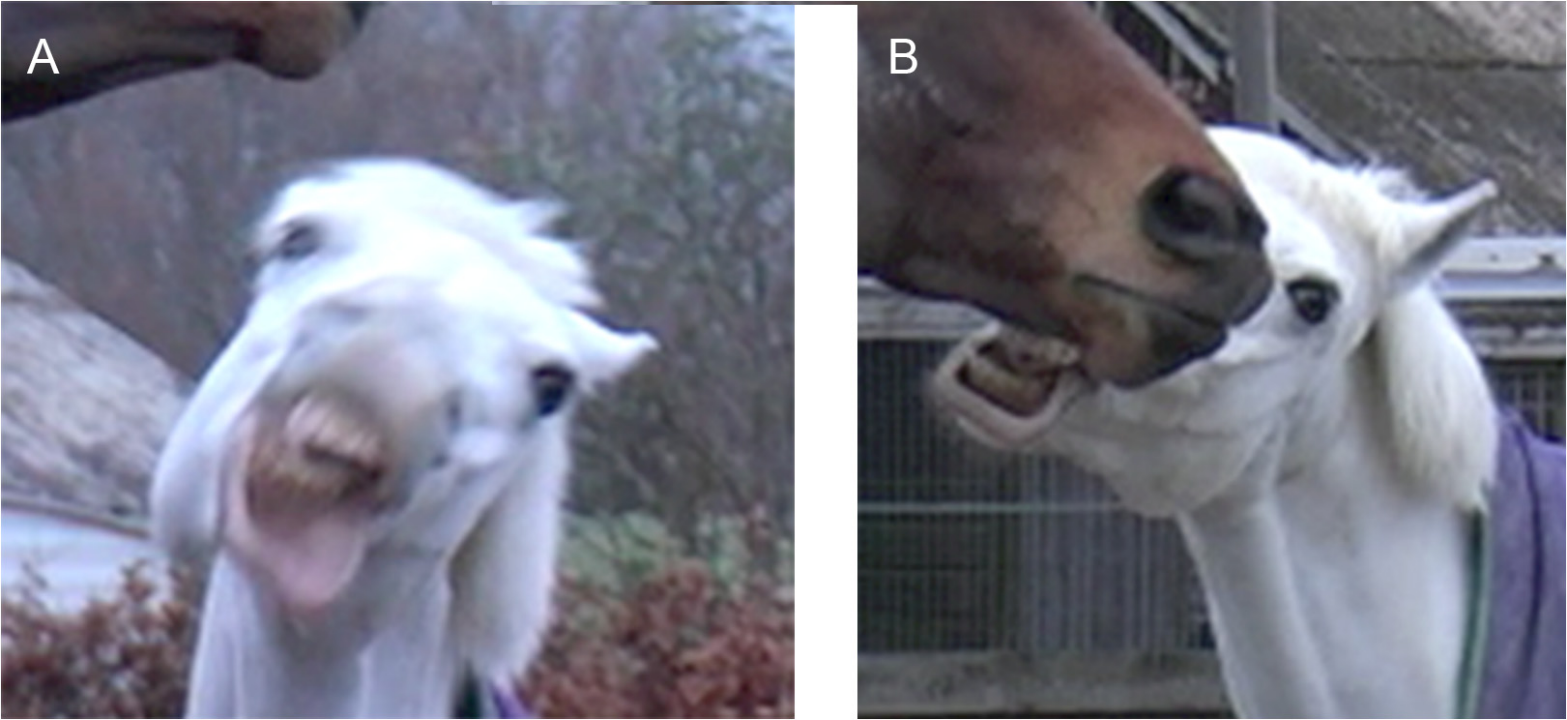
The lower lip when AU16, lower lip depressor, is acting in isolation (A) and in combination with AU17, chin raiser (B). Note the square shape of the lip and definition in the skin covering the mental region in combination AU16+17.

**B. Appearance changes due to AU16 and AU17:**
  1. The skin tightening action of 17 counteracts some of the lowering action on 16. This means that in combination 16 + 17, generally only the front section of the lower lip is pulled down, unlike in 16 alone where the whole lower lip may be pulled down and away from the jaw (see Fig 11).

2. This combined action of the muscles underpinning 16 and 17 may cause the lower lip to protrude as the skin over the chin is held tight while the lip is pulled down. The shape of the lower lip may be more square than when 16 is applied in isolation, due to the tightening action of 17 (see Fig 11).
3. At lower intensities the location of the movement can help to distinguish between AU16 and the combination of AU16 + 17. If the lip lowering seems to be coming from the insertion of the depressor labii inferioris then AU16 is acting alone, but if the lip lowering action seems to be only in the very front of the lip then 17 is acting with 16. For this reason it is much easier to distinguish between these actions from a ¾ or profile view. It can be difficult to distinguish between 16 alone and combination 16 + 17 from a frontal view.
4. When these action units are combined with very strong actions of AU27 be aware that the skin covering the chin will be stretched due to the jaw stretching action of 27, and this can interfere with the appearance changes of AU16 and/or 17. In these cases rely on the other appearance changes to help you establish whether AUs 16 and or 17 have occurred.
**C. Minimum criteria to code combination AU16 + 17:** both a lowering of the lower lip and a raising of the skin covering the mental region. See Fig 11 and S25 and S26 Videos for examples of this action.

#### Action Unit 18: Lip Pucker

AU 18 draws the lips medially, causing the lips to pucker and protrude. In humans this is generally seen in both lips, however in horses this action is mostly seen in the top lip only.

**A. Proposed muscular basis:** orbicularis oris muscle, incisivii labii muscle (Fig 1, see S2 Text for additional comments on these muscles).
**B. Appearance changes:**
  1. Pushes the lip forward and draws the lip medially, as if a drawstring were being pulled around the top lip.
  2. Shortens the mouth from a frontal view, making the mouth opening smaller and rounder, and the lip appear tight.
  3. Elongates the lip from a profile view, drawing the top lip rostrally.
  4. The lip becomes tense and moves away from the teeth. It may look as though it is hanging over the bottom lip.
  5. Horizontal wrinkles may appear along the face.
  6. There is no eversion of the lip in AU18. If this is seen then AU122, upper lip curl, or 18+122 must be scored.
  7. Very strong actions of AU18 may part the lips and draw the top lip up slightly.
  8. Often the lip can be drawn round to one side in this movement. This is due to AU18 acting more strongly on one side of the lip that the other. Do not score this as a unilateral action unless there is no trace of 18 on one side of the face.
**C. Minimum criteria to code AU18:** the top lip is tightened and drawn medially and forward away from the teeth. See S27, S28, and S29 Videos for examples of this action.
**D. Subtle differences:** the strands of muscle that underpin AU18 are also recruited in AU122, which leads to some shared appearance changes. The critical criterion to distinguish between these actions is whether there is any eversion of the top lip. If not, then score AU18; however, if there is score AU122. Also look for whether the action is isolated to the top lip (AU18), or whether it spreads further up the nose (AU122). AU18 and 122 can only be scored together if their actions are seen sequentially.

#### Action Unit 122: Upper Lip Curl

AU122 resembles AU22, the Lip Funneler, which is seen in humans and chimpanzees. However, in horses this movement has a partially different muscular basis and involves the transverse nasi and levator labii superioris muscles in addition to the orbicularis oris muscle. In AU122 the lip is pushed forward and curls/flares. By definition this must part the lips, and so must always be scored with an AU25, lips part, and this action is usually only seen in the top lip.

**Fig 12:**
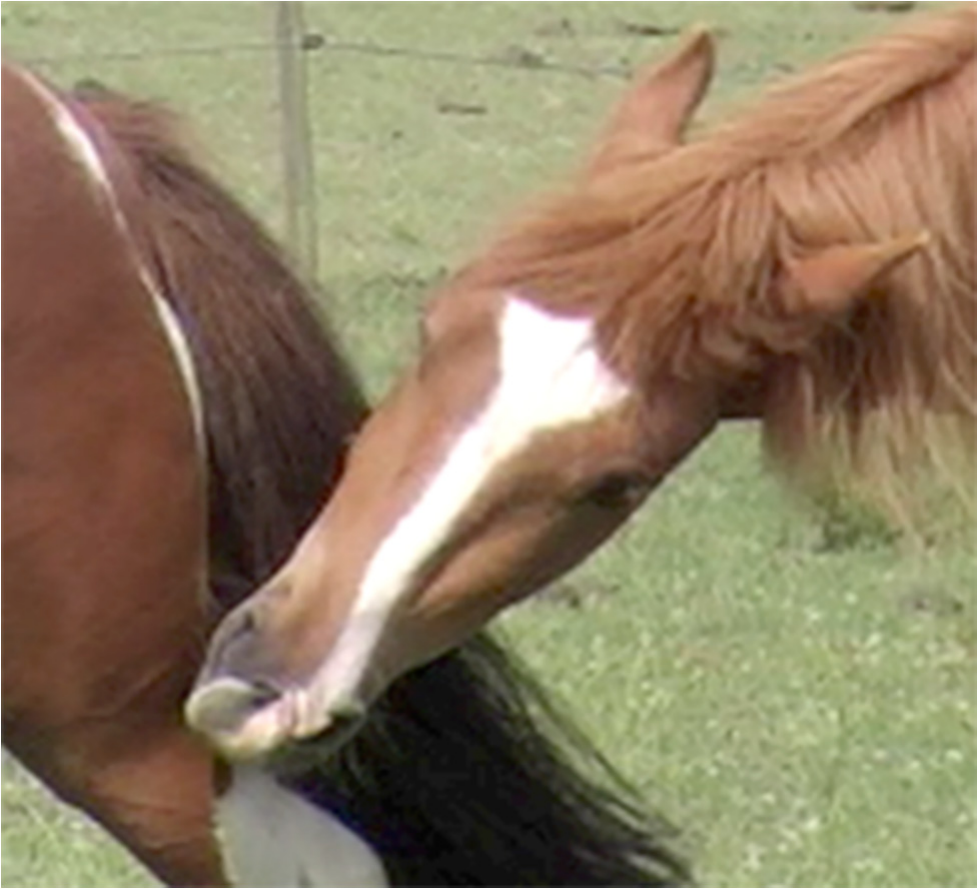
An example of the wrinkles seen with AU122, upper lip curl. These wrinkles are also characteristic of AU10, upper lip raiser, however in AU10 there is no puckering or eversion of the lip.

**A. Proposed muscular basis:** levator labii superioris muscle, transverse nasi labii muscle, orbicularis oris muscle (Fig 1, see S2 Text for additional comments on these muscles).
**B. Appearance changes:**
  1. The upper lip everts and curls up.
  2. The action is focused on the medial portion of the upper lip.
  3. The upper lip will pucker, sharing some of the appearance changes with AU18 – see the section on subtle differences for help distinguishing the two actions.
  4. There is distinctive wrinkling across the nose, and along the side on the nose (see Fig 12). This wrinkling is also seen in AU10, upper lip raiser, see the section on subtle differences for help distinguishing the two actions.

5. This action may be held for a long time.

**C. Minimum criteria to code AU122:** the lips must part and the upper lip must curl up. See S30, S31, and S32 Videos for examples of this action.
**D. Subtle differences:** AUs122 and 10, upper lip raiser, are difficult to distinguish at low intensities. Look at whether the upper lip puckers and moves away from the gum; if so code AU122, but if not then code AU10. At higher intensities the upper lip curl/eversion is important to distinguish AU122 from AU10, in which the upper lip only lifts and does not curl. Similarly, weak actions of AUs122 and 18, lip pucker, are difficult to discriminate, as both involve lip puckering. Watch for the pulling of the lip from higher up the nose, in between the nostrils, and transverse wrinkles across the nose that only occur in AU122 and not 18. If the upper lip is puckered but not curled up then code AU18. Be aware that these actions may often occur in rapid succession (e.g. S32 Video).

### Action Unit 24: Lip Presser

**A. Proposed muscular basis:** orbicularis oris muscle (Fig 1, see S2 Text for additional comments on this muscle).
**B. Appearance changes:**
  1. Presses the lips together, without pushing up the skin covering the mental region (if this is seen also code AU17, chin raiser).
  2. Lowers the upper lip and raises the lower lip to a small extent.
  3. Tightens and narrows the lips.
  4. May cause a bulging of the skin above the upper and/or lower lip.
  5. Both lips are drawn in towards the mouth.
**C. Minimum criteria to code AU24:** the lips must be pulled in and pressed together. See S33 and S34 Video for examples of this action.
**D. Subtle differences:** the pressing together in AU24 is caused by a muscular movement, and not just the movement of the jaws, e.g. in chewing. If lower lip alone is moved up (and this pushes up the top lip too) then code AU17. AU24 and 17 can occur together, in this case look for action further down the lower lip, in the skin covering the mental region (S34 Video).

#### Action Units 25, 26, and 27: Lips Part, Jaw Drop, and Mouth Stretch

These three AUs describe mouth opening, including separation of the lips and the teeth. Although there could be an argument for classifying these as Action Descriptors rather than Action Units, we have described them as Action Units here to remain consistent with previous FACS. AU25 denotes separation of the lips, so can be coded independently or with AU26 or 27. AU26 and 27 both refer to parting of the jaw; and so can be coded with or without 25. However, AU26 and 27 are mutually exclusive as they designate different types of jaw opening, and so AU26 and 27 must not both be coded for the same movement. Although AU26 and 27 both code opening of the mouth, they refer to different actions. AU26 refers to the dropping of the lower jaw that is caused by relaxation of muscles. However, AU27 refers to a more purposeful movement where the jaw is stretched apart by contraction of muscles.

### Action Unit 25: Lips Part

**A. Proposed muscular basis:** orbicularis oris, levator labii superioris, levator nasolabialis, depressor labii, transverse nasi, zygomaticus muscles, or the action may not be muscular but may be caused by opening the jaw (Fig 1, see S2 Text for additional comments on this muscle).
**B. Appearance changes:**
  1. The lips are separated at any point around the mouth.
  2. There is a gap seen between the top lip and bottom lip.
  3. In stronger actions the gums or teeth may be visible.
**C. Minimum criteria to code AU25:** the lips must be seen to part at some point. See S35 Video for an example of this action.

### Action Unit 26: Jaw Drop

**A. Proposed muscular basis:** this movement is not caused by the facial muscles, but by muscles such as the masseter.
**B. Appearance changes:**
  1. The lower jaw is lowered and teeth separation can be clearly seen or at least inferred.
  2. The movement is relaxed and the jaw does not open so much that it is causing an obvious stretch.
  3. Although AU26 can be coded with AU25 it does not have to be. If there are signs of jaw lowering (i.e. you can see the skin move) without the lips parting, then score AU26 alone.
**C. Minimum criteria to code AU26:** there must be movement of lower jaw either seen through movement of the overlying skin, or teeth separation. See S36 Video for an example of this action.

### Action Unit 27: Mouth Stretch

**A. Proposed muscular basis:** this movement is not caused by the facial muscles, but by muscles such as the masseter.
**B. Appearance changes:**
  1. The lower jaw is pulled down and teeth separation can be clearly seen.
  2. The movement is purposeful and the mouth appears to be clearly stretched open, rather than the more relaxed jaw lowering in AU26.
  3. It is likely that AU27 will be coded with AU25, as the degree of jaw opening in AU27 is usually strong enough to part the lips.
  4. The lips may retract or change shape to accommodate the degree of mouth opening.
**C. Minimum criteria to code AU27:** the jaw must be parted and this action must be through an obvious mouth stretch. See S37 Video for an example of this action.

### Ear Action Descriptors

Ear actions do not feature in many primate FACS, as the auricular (ear) muscles of many the primates are gracile and vestigial. Rhesus macaques are an exception where ear action descriptors have been described [8, 10], as they are for the domestic dog [12] and cat [13].

Horses have an extremely complex and robust set of auricular muscles that allow a great range and specificity of movement. However, the ears of horses are obvious and large, with little variation in the shape of the pinna (external ear), making it relatively easy to code ear movements. Often both of the ears are visible simultaneously, unlike the other movements described in EquiFACS where one side of the face is likely to be obscured. If one ear is out of sight it cannot be assumed that any action seen in the visible ear is bilateral. The ears can, and often do, move independently, and so it should be carefully noted if a movement is unilateral or if one ear is out of sight (AD 75).

**Fig 13:**
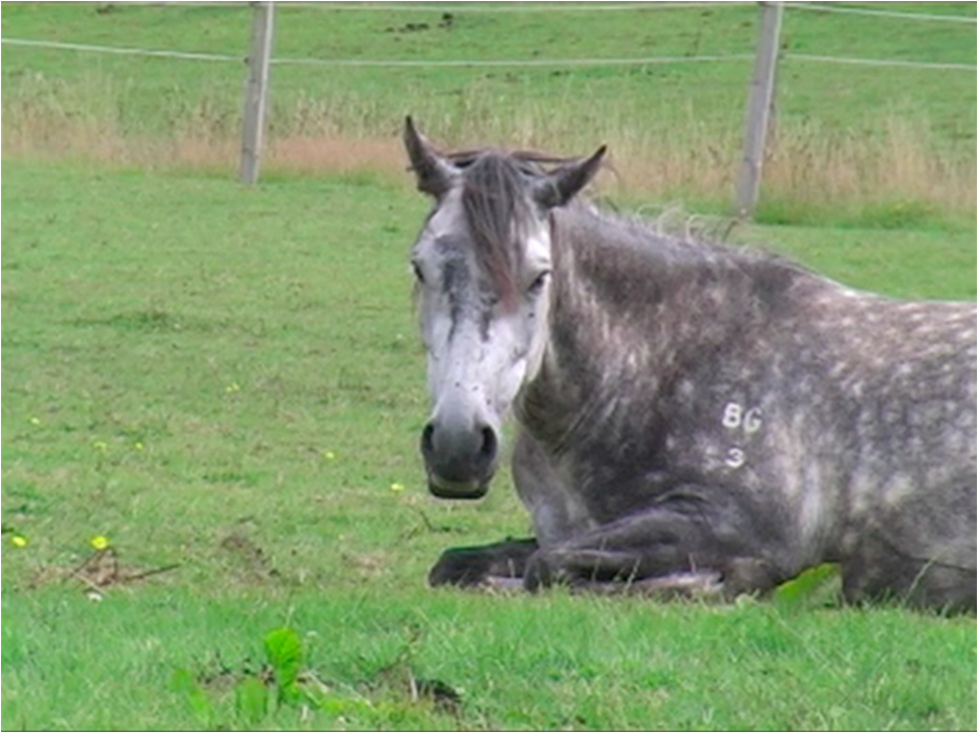
The general neutral ear position of a horse, although there is some individual variation.

Action descriptors are given rather than specific action units, as due to the complex muscular network around the auricle it is impossible to know the exact basis of the ear movements. For this reason section **A. Proposed Muscular Basis,** is omitted from the descriptions below. Before coding ear movements in horses, a neutral position for each individual must be determined. This helps to distinguish between genuine ear movements and a release of a previous movement that is returning the ears to neutral. The ideal time to obtain sight of a neutral position is when the horse is resting (e.g. Fig 13).

### Ear Action Descriptor 101: Ears Forward

**B. Appearance changes:**
  1. The ear(s) are turned or swivel forward (rostrally).
  2. From a frontal view the amount of inner ear seen increases.
  3. From a profile view the amount of inner ear seen may decrease (although this depends on the strength of the movement).
  4. The distance between the tips of the pinna decreases.
**C. Minimum criteria to code EAD101:** a rostral rotation of the pinna. See S38 and S39 Videos for examples of this action.

### Ear Action Descriptor 102: Ear Adductor

**B. Appearance changes:**
  1. The ear(s) are pulled towards the midline (i.e. adducted).
  2. The distance between ears decreases, this is particularly obvious in the tips of the pinna.
  3. If viewed in profile, then the amount of inner ear/ear opening visible will increase.
  4. This is a separate movement to EAD101 (although EAD101 and EAD102 may be coded together).
**C. Minimum criteria to code EAD102:** movement of at least one ear towards the midline. See S40 Video for an example of this action.
**D. Subtle differences:** in EAD101 the ears may become closer together as the ears are brought round and forward in a rotational movement. However, unless the movement clearly pulls the ears in towards the midline, do not code EAD102. Only use EAD102 when there is a movement in towards the midline that draws the medial side of the pinna to a more acute angle on the head, and when this movement cannot be explained by a rotational movement alone. Often when EAD101 and EAD102 are present together the sequential application of these actions is visible.

### Ear Action Descriptor 103: Ear Flattener

**Fig 14:**
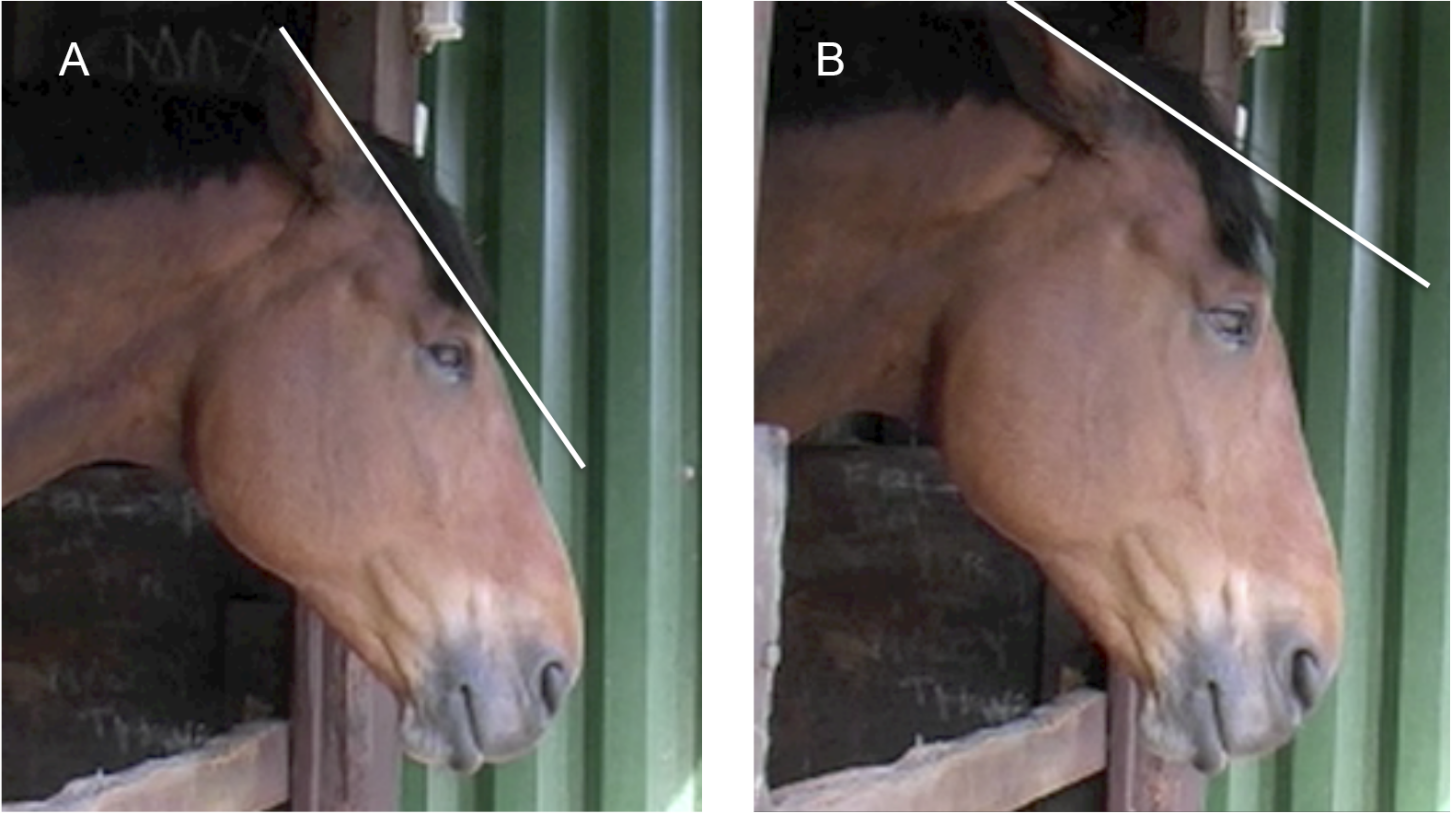
The starting ear position (A) and then with EAD103, ear flattener, applied (B). Note how the angle of the ears to the front of the face alters.

**B. Appearance changes:**
  1. The ear(s) are flattened and abducted (Fig 14).

**Fig 15:**
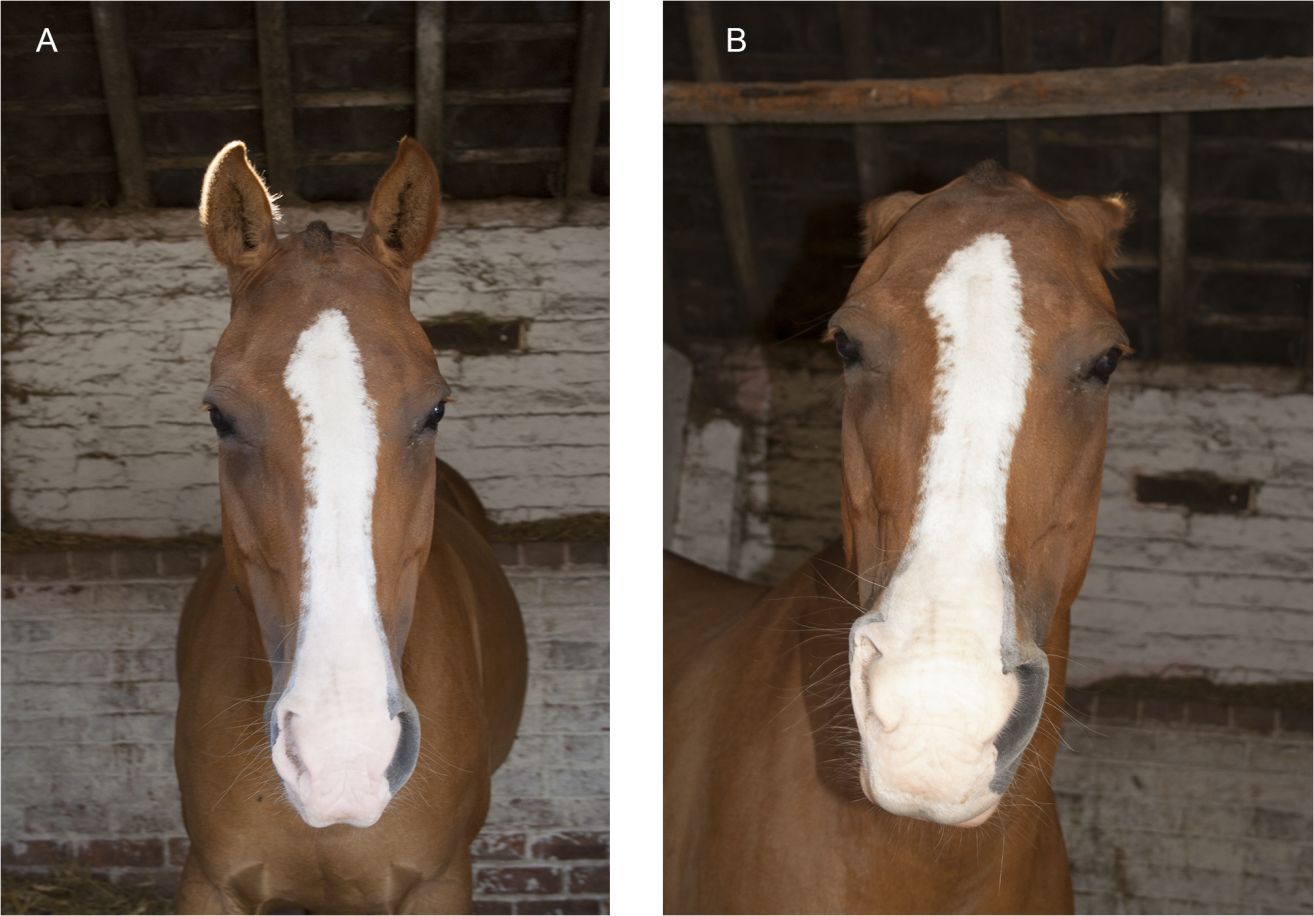
The starting ear position (A) and then with EAD103, ear flattener, and EAD104, ear rotator, applied (B). An example of how the ears can almost disappear in a frontal view of a strong action.

2. From a profile view the amount of inner ear visible will decrease and the angle of the ears relative to the midline will increase.
3. This is often (although not always) seen with EAD104, ear rotator. In this case the combined action of EAD103+EAD104 alters the appearance of the flattening movement compared to when EAD103 is acting alone.
4. When applied with EAD104 the angle of the ears to the front of the nose will increase (Fig 14) and in a frontal profile, the ear(s) may disappear from view behind the head (Fig 15).

**C. Minimum criteria to code EAD103:** the ears are pulled caudally. See S41 and S42 Videos for examples of this action.

### Ear Action Descriptor 104: Ear Rotator

**B. Appearance changes:**
  1. The ears are rotated laterally and dorsal/caudally. The opening of the inner ear is turned outwards.
  2. This is the opposing movement to EAD101, ears forward, and the ears will swivel in the opposite direction.
  3. Ensure that this is not confused with EAD103, ear flattener. EAD104 is a rotational movement, whereas EAD103 flattens the ears to the head/neck. The two actions may happen simultaneously.
  C. Minimum criteria to code EAD104: the pinna rotates caudally. See S43 Video for an example of this action.

### Miscellaneous Actions and Supplementary Codes

A variety of miscellaneous actions and supplementary codes are given in S5 Text. Whilst these do not describe facial movements, they denote actions that can influence the coding of facial expression.

## Conclusions

The study of complex behaviours, such as facial expressions, would be limited without standardised systems that allow quantification of behaviour and cross-species comparisons. Facial Action Coding Systems (FACS) provide a means of addressing this problem, and by developing a FACS for the domestic horse (EquiFACS) we have produced a tool that allows the systematic recording of horse facial expressions in any context, with the potential to make direct comparisons across species. EquiFACS is an anatomically based, objective system that contains all the facial actions horses can possibly produce, making it suitable for many different research questions.

Horses have a rich facial repertoire, with 17 defined Action Units (AUs). Whilst this was less than the number of AUs humans display (27), it was slightly more than most other animals that FACS have been developed for (chimpanzees, 13, rhesus macaques, 13, orang-utans, 15, Hylobatids, 16, dogs, 16), with only cats displaying a larger facial repertoire (21, largely due to the extensive whisker and ear movements). The potential to make such cross species comparisons can enhance our understanding of the meaning, function, and evolution of communicative behaviour [14, 44]. In fact, many of the horse AUs were similar to the facial movements seen in other animals, including humans, chimpanzees, cats, and dogs. It was previously thought that humans possessed the most complex repertoire of facial expressions and that, in phylogenetic terms, the further away an animal was from humans, the more rudimentary their use of facial expressions would be. However, through the development of EquiFACS it is apparent that horses also have an extensive range of facial movements, sharing many Action Units with humans and other animals. This contributes to a growing body of evidence suggesting that the evolution of facial expressions was not driven entirely by phylogenetic pressures, but that other, socio-ecological factors had a significant influence [30, 35].

EquiFACS also provides those working in the horse community with a standardised language through which information can be shared, facilitating the investigation of questions relevant to horse management and welfare. It should be noted that whilst some of the video examples given with this manuscript contain recognisable horse behaviour (e.g. flehmen, S25 and S30 Videos), when coding facial expressions it is important not to try and immediately assess the overall behaviour, but instead to focus on describing the individual facial movements. One of the key actions seen in flehmen (AU122, the upper lip curl) has also been recorded in some pain expressions of the horse [45, 46], and yet is also seen in other, unrelated contexts (see S31 Video). A potential application of EquiFACS would be to establish whether there are particular configurations of facial movements displayed alongside the upper lip curl (AU122), which help distinguish the different contexts.

While recent work has suggested that horses use apparently complex facial expressions [28, 47], and that certain facial movements are associated with pain in domestic horses [2, 3], until now the full capacity of horse facial expressions to convey a range of information has been largely overlooked. In particular, no studies have yet investigated whether there are facial expressions associated with positive experiences in horses–a critical yet poorly understood aspect of animal welfare [48]. EquiFACS can be applied to address this gap, with the potential to greatly facilitate future studies of horse welfare as well as extending our knowledge of equine communication and cognition.

## Supporting Information

S1 Fig The facial mask from the right side of the face. The suffix ‘m.’ refers to muscle. OOM: orbicularis oris muscle. CN7: cranial nerve 7. LAOF m.: levator annuli oris fascialis muscle. OOC: orbicularis occuli muscle. SAP major m.: scutulo-auriculartis profundus major muscle. ZAm.: zygomatico-auricularis muscle. FS m. temporal part: frontoscutularis muscle temporal part. DLI m.: depressor labii inferioris muscle.
(TIF)

S2 Fig The muscles in the lips and midface of the horse. The suffix ‘m.’ refers to muscle. OOM: orbicularis oris muscle. CN7: cranial nerve 7. LAOF m.: levator annuli oris fascialis muscle.
(TIF)

S3 Fig The muscles in the lower face of the horse. The suffix ‘m.’ refers to muscle. OOM: orbicularis oris muscle. DLI m.: depressor labii inferioris muscle.
(TIF)

S4 Fig The muscles around the ear of the horse. The suffix ‘m.’ refers to muscle. OOC: orbicularis occuli muscle. FS m. temporal part: frontoscutularis muscle temporal part. SAP minor m.: scutulo-auriculartis profundus minor muscle. SAP major m.: scutulo-auriculartis profundus major muscle. PA m.: partoidoauricularis muscle. ZAm.: zygomatico-auricularis muscle.
(TIF)

S1 Text Detailed dissection protocol. (DOCX)

S2 Text Detailed descriptions of the facial muscles of the horse. (DOCX)

S3 Text Coding instructions. (DOCX)

S4 Text Calculating the proportion of visible eye-white. (DOCX)

S5 Text Miscellaneous actions and supplementary codes. (DOCX)

S1 Video AU101, inner brow raiser. (MOV)

S2 Video AU101, inner brow raiser. (MOV)

S3 Video AU143, eye closure. (MOV)

S4 Video AU143, eye closure. (MOV)

S5 Video AU145, blink. (MOV)

S6 Video AU47, half blink. (MOV)

S7 Video AU47, half blink, followed by release of AU101, inner brow raiser. (MOV)

S8 Video AU5, lid raiser, and AD1, eye white increase. (MOV)

S9 Video AU5, lid raiser, and AD1, eye white increase. (MOV)

S10 Video AD1, eye white increase, in isolation. (MOV)

S11 Video AU10, upper lip raiser. NB numerous other AUs are present in this video: specifically AU18, lip pucker, and AUH13, nostril lift, act on the upper lip at the beginning of the clip, prior to the start of the AU10 movement.
(MOV)

S12 Video AU10, upper lip raiser seen in the lighter chestnut horse. (MOV)

S13 Video AU12, lip corner puller. (MOV)

S14 Video AU12, lip corner puller, seen repeatedly. NB AU113, sharp lip puller, (plus other AUs) are also present—see section with AU113 for advice about how to identify when AU12 and AU1113 are acting simultaneously.
(MOV)

S15 Video AU12, lip corner puller, without AU25, lips part, and AU27, mouth stretch. (MOV)

S16 Video AU113, sharp lip puller. (MOV)

S17 Video AU113, sharp lip puller. (MOV)

S18 Video AUH13, nostril lift. (MOV)

S19 Video AUH13, nostril lift, seen repeatedly from a frontal view. (MOV)

S20 Video AU16, lower lip depressor. (MOV)

S21 Video AU16, lower lip depressor, seen in the horse on the right. (MOV)

S22 Video AD160, lower lip relax. (MOV)

S23 Video AU17, chin raiser, seen in the horse on the right. (MOV)

S24 Video AU17, chin raiser, seen in the horse on the left. (MOV)

S25 Video AU16, lower lip depressor, followed by multiple actions of combination AU16+17, chin raiser. (MOV)

S26 Video AU16, lower lip depressor, alone followed by 16 and 17, chin raiser, acting together, show in the horse on the right. (MOV)

S27 Video AU18, lip pucker, in the grey (white) horse. (MOV)

S28 Video AU18, lip pucker. (MOV)

S29 Video AU18, lip pucker, from a frontal view. Note that although the white hair on the upper lip can make it difficult to establish if AU18 has occurred, the horizontal wrinkles across the nose identify the action.
(MOV)

S30 Video AU122, upper lip curl. (MOV)

S31 Video AU122, upper lip curl, seen in the horse on the left. (MOV)

S32 Video AU10 turning rapidly into AU122 seen in the horse on the left. Note the distinguishing point between these actions is when the upper lip protrudes from the upper jaw.
(MOV)

S33 Video AU24, lip presser. (MOV)

S34 Video AU24, lip presser, with AU17, chin raiser. (MOV)

S35 Video AU25, lips part, seen with other AUs (AU16, AU17, AU16+17, AU122, AD1, EAD102). (MOV)

S36 Video AU26, jaw drop, with AU25, lips part, seen in the horse on the right. N.B. This is roughly the limit of jaw opening that would be scored as AU26. Generally, if the jaw were opened more than this it would be scored AU27, mouth stretch.
(MOV)

S37 Video AU27, mouth stretch, with AU25, lips part, seen in the horse on the left. (MOV)

S38 Video EAD101, ears forward. (MOV)

S39 Video L EAD101, ears forward seen only in the left ear. (MOV)

S40 Video L EAD102, ear adductor. NB EAD 104 (R) is also seen.
(MOV)

S41 Video EAD103, ear flattener, with EAD104, ear rotator, followed by an action of EAD103 alone. (MOV)

S42 Video EAD103, ear flattener, with EAD104, ear rotator, seen in the grey (white) horse. (MOV)

S43 Video EAD104, ear rotator. (MOV)

S44 Video AD29, jaw thrust, and AD30, jaw sideways, seen in the horse on the right. (MOV)

S45 Video AD133, blow. (MOV)

S46 Video AD38, nostril dilator. (MOV)

S47 Video AD 38, nostril dilator, with septum lift (appearance change 3). (MOV)

S48 Video AD55, head tilt left, seen in the horse on the right of the image, and AD56, head tilt right, seen in the horse on the left of the image. (MOV)

S49 Video AD57, nose forward, seen in the grey (white) horse. (MOV)

S50 Video AD58, nose back, seen in the grey (white) horse. (MOV)
