# Correction: EquiFACS: The Equine Facial Action Coding System

**DOI:** 10.1371/journal.pone.0137818

**Published:** 2015-09-03

**Authors:** Jen Wathan, Anne M. Burrows, Bridget M. Waller, Karen McComb

The following information is missing from the Funding section: the Biotechnology and Biological Sciences Research Council grant no. BB/D526896/1.

The complete, correct Funding statement is: JW was supported by a studentship from the Biotechnology and Biological Sciences Research Council, grant no. BB/D526896/1 (BBSRC—www.bbsrc.ac.uk) and the University of Sussex, and also an Alumni Travel Grant from the University of Sussex (www.sussex.ac.uk). KM was supported by a Leverhulme Trust Research Grant number RPG-2013-069 (www.leverhulme.ac.uk). The funders had no role in study design, data collection and analysis, decision to publish, or preparation of the manuscript.

In addition, the affiliations for the second and third authors are incorrectly switched. Anne M. Burrows is not affiliated with #2 but with #3, Department of Physical Therapy, Duquesne University, Pittsburgh, Pennsylvania, United States of America; Bridget M. Waller is not affiliated with #3 but with #2, Centre for Comparative and Evolutionary Psychology, Department of Psychology, University of Portsmouth, Portsmouth, United Kingdom.
