# Supplementary figures and images for: EquiFACS: The Equine Facial Action Coding System
Source: PLoS One. 2015 Aug 5;10(8):e0131738. doi: 10.1371/journal.pone.0131738 (PMC4526551; doi:10.1371/journal.pone.0131738)

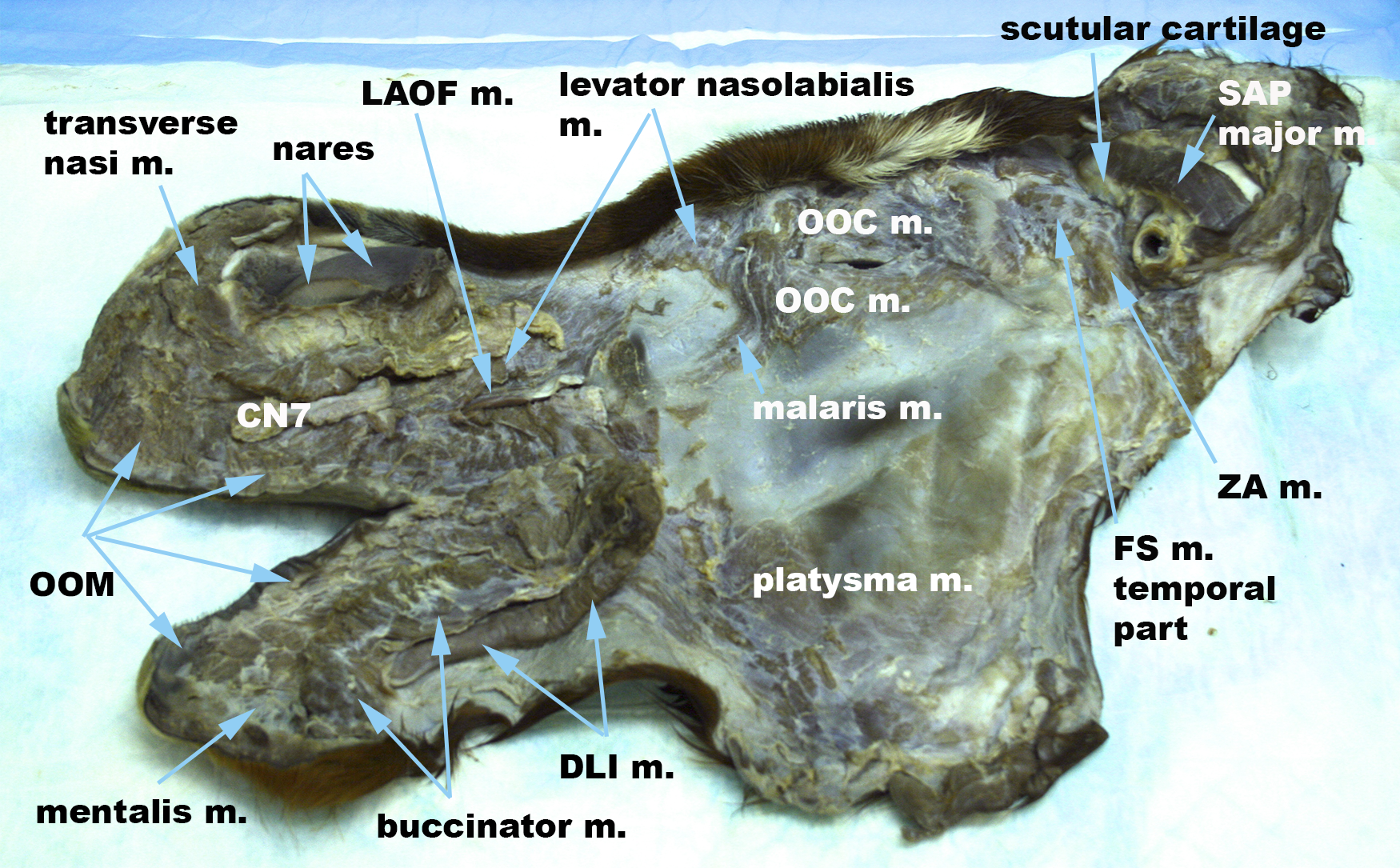

Supplement: S1 Fig — The suffix ‘m.’ refers to muscle. OOM: orbicularis oris muscle. CN7: cranial nerve 7. LAOF m.: levator annuli oris fascialis muscle. OOC: orbicularis occuli muscle. SAP major m.: scutulo-auriculartis profundus major muscle. ZAm.: zygomatico-auricularis muscle. FS m. temporal part: frontoscutularis muscle temporal part. DLI m.: depressor labii inferioris muscle. (TIF) [file pone.0131738.s001.tif]

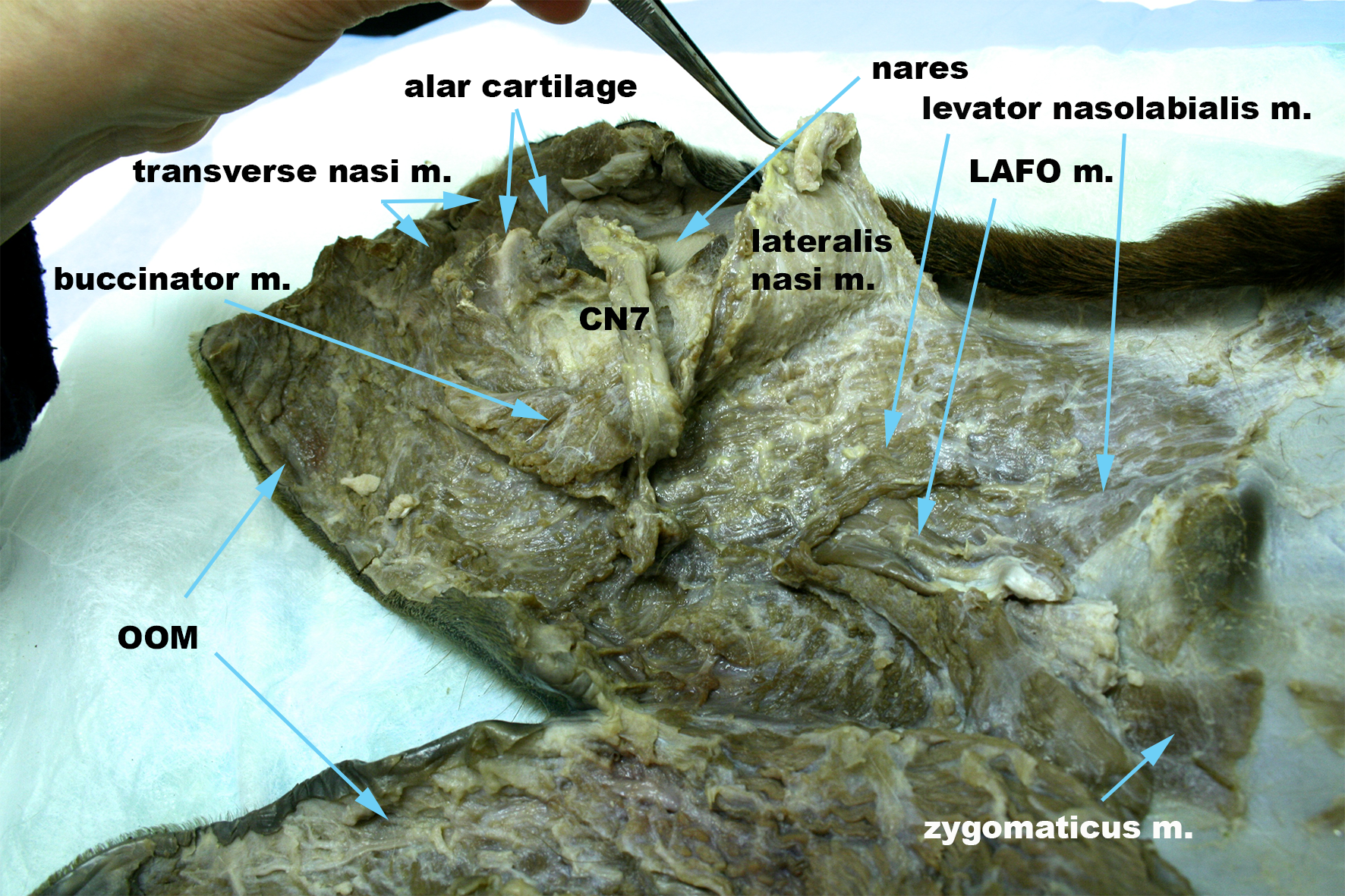

Supplement: S2 Fig — The suffix ‘m.’ refers to muscle. OOM: orbicularis oris muscle. CN7: cranial nerve 7. LAOF m.: levator annuli oris fascialis muscle. (TIF) [file pone.0131738.s002.tif]

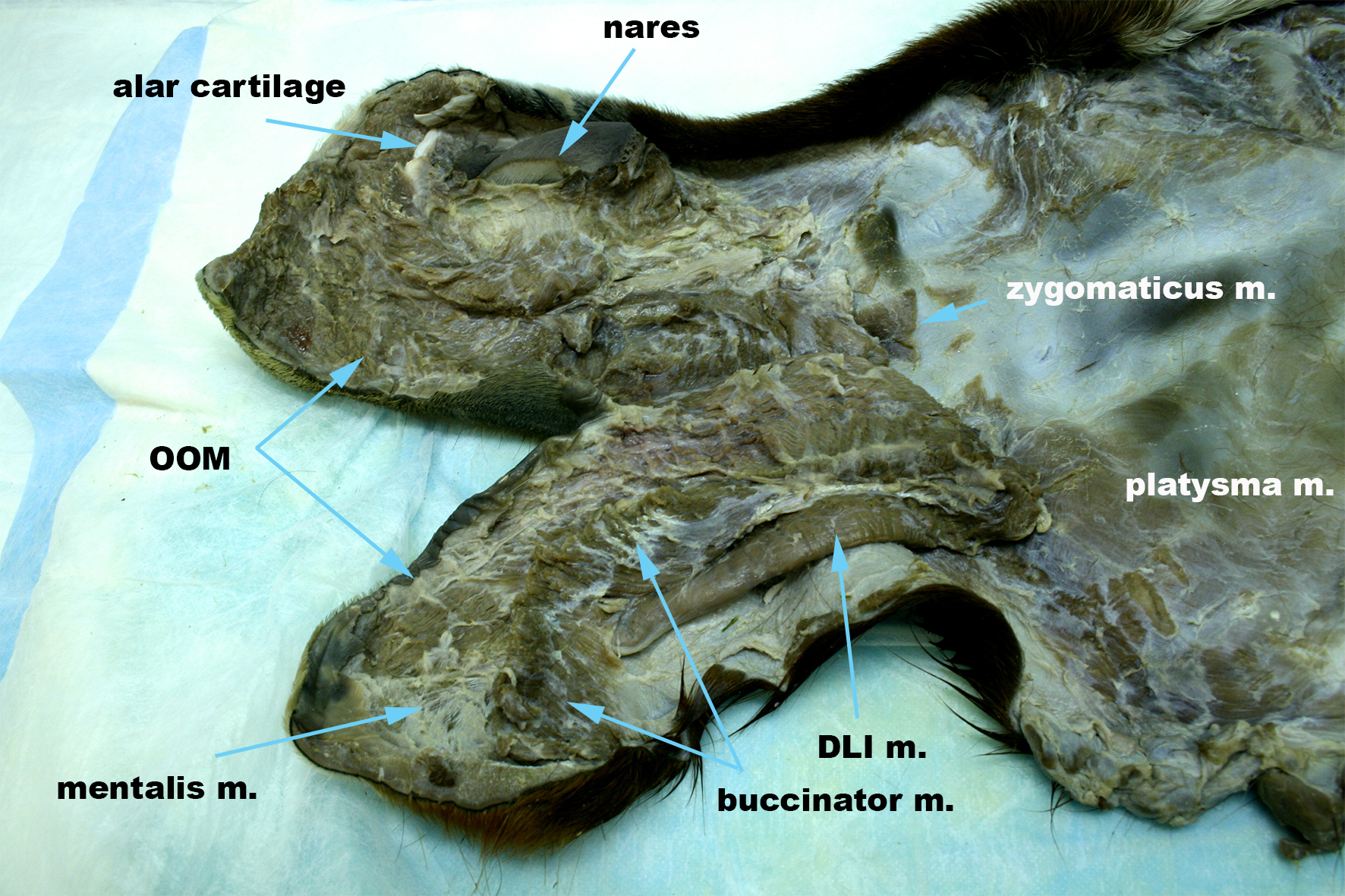

Supplement: S3 Fig — The suffix ‘m.’ refers to muscle. OOM: orbicularis oris muscle. DLI m.: depressor labii inferioris muscle. (TIF) [file pone.0131738.s003.tif]

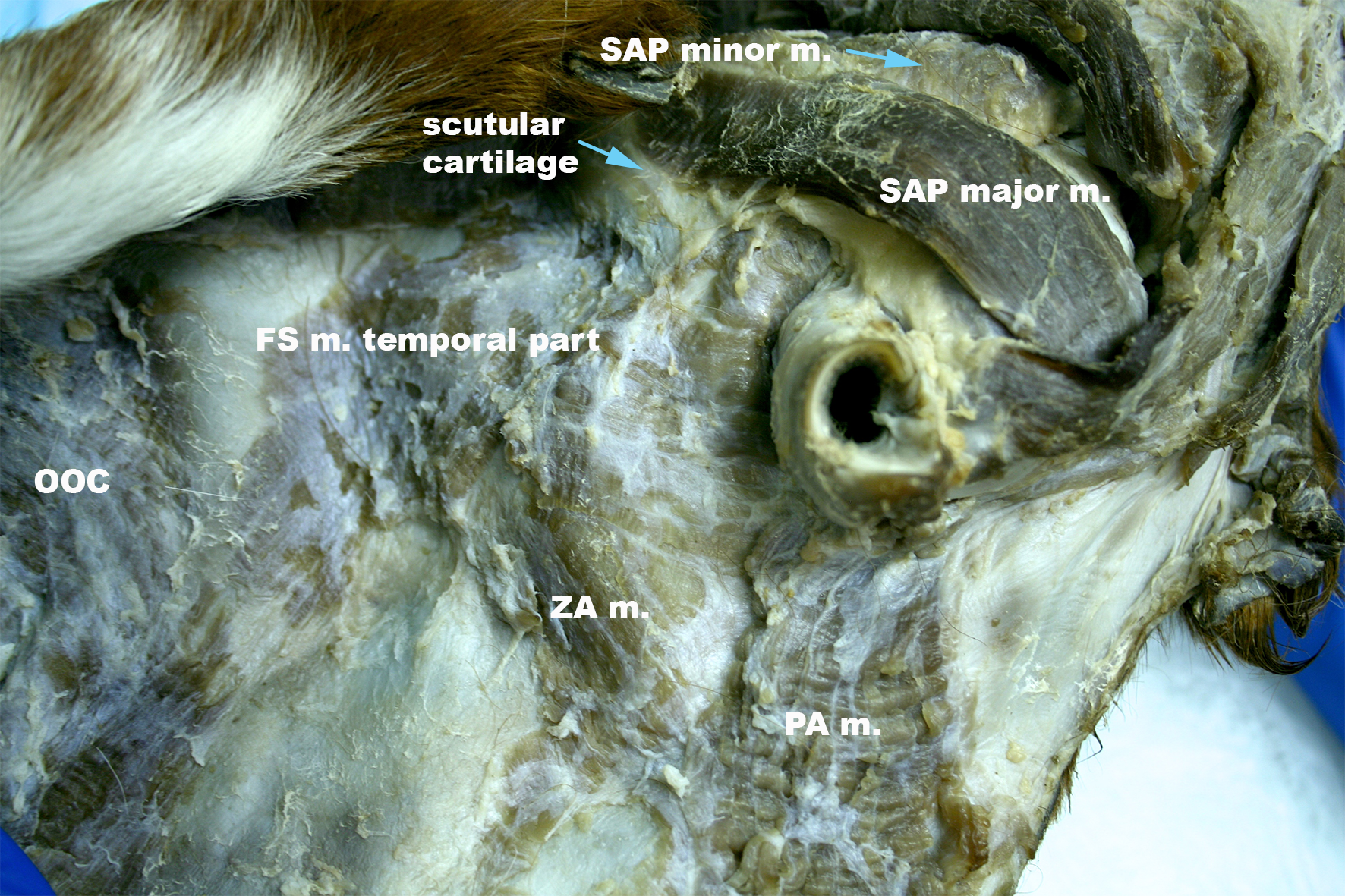

Supplement: S4 Fig — The suffix ‘m.’ refers to muscle. OOC: orbicularis occuli muscle. FS m. temporal part: frontoscutularis muscle temporal part. SAP minor m.: scutulo-auriculartis profundus minor muscle. SAP major m.: scutulo-auriculartis profundus major muscle. PA m.: partoidoauricularis muscle. ZAm.: zygomatico-auricularis muscle. (TIF) [file pone.0131738.s004.tif]
