# Supplementary material for: EquiFACS: The Equine Facial Action Coding System
Source: PLoS One. 2015 Aug 5;10(8):e0131738. doi: 10.1371/journal.pone.0131738 (PMC4526551; doi:10.1371/journal.pone.0131738)
Supplement: S1 Text — (DOCX) [file pone.0131738.s005.docx]

**Detailed dissection protocol**

Demographic information for the specimen was not available, however age was approximated from the teeth demonstrating that the specimen was an adult (circa 7 years). The tongue and larynx were removed, however the brain was still intact so the muscles of the cranial vault were undisturbed. The head was disarticulated from the neck at the atlanto-occipital joint. Where the head had been detached some of the cervical portion of the fascia was missing so the full extents of some of the ear muscles were unavailable. The head was obtained frozen and thawed in phosphate buffered saline solution prior to dissection.

The skin, superficial fasciae, and facial musculature were separated from the more deeply located muscles (the masseter and temporalis muscles) with a #21 scalpel blade and a variety of dissection tools. The right side of the face and superficial fascia were removed from the head first, beginning at the mandibular skin flap created when the head was removed from the body. The skin was removed from the inferior border of the mandible, and then a midline incision was made through the lower lip and the frontal and parietal regions. Finally the muscles of the external ear were released, and the external ear was removed with the skin. The levator labii superios proprius muscle was the only facial muscle to be left on the head. This procedure was then repeated for the left side of the face.

Care was taken to remove as much of the facial musculature as possible with the skin and superficial fasciae leaving behind only the bony attachments. This process created a “facial mask” for both the right and left side of the face that was separate from the skull and held all of the facial muscles except for the levator labii superious muscle, which was left behind with the skull. On the left side of the face the muscles of the lip (orbicularis oris, buccinator, and mentalis muscles) were also left on the skull, to give an alternative view of the relationships between the muscles and their relationship to the skull.

Once removed the face masks were examined before being fixed in 5% formalin solution, and initial impressions were recorded. Once fixed the right face mask was brought out and allowed to air dry for 30 minutes to allow the best differentiation between the tissues, before the connective tissue was released from the musculature. This left the facial muscles discernable from the surrounding muscles and fasciae (see [[1-4](#_ENREF_1)]).

References

1. Burrows AM, Diogo R, Waller BM, Bonar CJ, Liebal K. Evolution of the Muscles of Facial Expression in a Monogamous Ape: Evaluating the Relative Influences of Ecological and Phylogenetic Factors in Hylobatids. The Anatomical Record: Advances in Integrative Anatomy and Evolutionary Biology. 2011;294(4):645-63. doi: 10.1002/ar.21355.

2. Burrows AM, Smith TD. Muscles of facial expression in Otolemur, with a comparison to lemuroidea. Anat Rec A Discov Mol Cell Evol Biol. 2003;274(1):827-36. Epub 2003/08/19. doi: 10.1002/ar.a.10093. PubMed PMID: 12923893.

3. Burrows AM, Waller BM, Parr LA. Facial musculature in the rhesus macaque (Macaca mulatta): evolutionary and functional contexts with comparisons to chimpanzees and humans. Journal of Anatomy. 2009;215(3):320-34. doi: 10.1111/j.1469-7580.2009.01113.x.

4. Burrows AM, Waller BM, Parr LA, Bonar CJ. Muscles of facial expression in the chimpanzee (Pan troglodytes): descriptive, comparative and phylogenetic contexts. Journal of Anatomy. 2006;208(2):153-67. doi: 10.1111/j.1469-7580.2006.00523.x.
