# Supplementary material for: EquiFACS: The Equine Facial Action Coding System
Source: PLoS One. 2015 Aug 5;10(8):e0131738. doi: 10.1371/journal.pone.0131738 (PMC4526551; doi:10.1371/journal.pone.0131738)
Supplement: S2 Text — (DOCX) [file pone.0131738.s006.docx]

**Detailed descriptions of the facial muscles**

See Figure 1 for an illustration of the facial muscles.

**The eye region**

**Corrugator Supercilii** **Muscle**

The corrugator supercilii muscle is a thin, small but distinct muscle immediately deep to the skin. It is attached to the root of the supraorbital process and to the skin of the upper eyelid, blending with the fibres of the orbicularis oculi muscle [[1](#_ENREF_1)]. Although this muscle was reported in some texts (e.g. [[1](#_ENREF_1)]) it was overlooked in others [[2](#_ENREF_2)].

**Levator Anguli Oculi Medialis** **Muscle**

In our specimen this muscle presented as a robust band of longitudinal fibres within the orbicularis oculi muscle, rather than a distinct, separate muscle. The fibres attached to the base of the zygomatic process of the frontal bone and to the medial surface of the skin of the upper eyelid. This description was consistent with previous reports [[2](#_ENREF_2)], however this muscle was omitted from other texts [[1](#_ENREF_1)].

**Orbicularis Oculi Muscle**

This is a flat, elliptical sphincter muscle attached to the skin of the eyelids. This muscle is thin, but extensive, similar to the orbicularis oculi muscle in primates [[3-6](#_ENREF_3)]. The portion of orbicularis oculi muscle surrounding the upper eyelid was substantially wider than the portion of the lower eyelid. The orbicularis oculi muscle is mostly attached to the skin of the eyelids, but some bundles are attached to the palpebral ligament at the medial canthus and to the lacrimal bone [[1](#_ENREF_1), [2](#_ENREF_2)].

**Levator Palpebrae Superioris Muscle**

This muscle technically belongs to the upper eyelid. We did not dissect the eye itself, and so could not locate this muscle; however, due to its presence in all investigated mammals, we expect that it would have been present [[7](#_ENREF_7)].

**Levator Labii Superioris Alaeque Nasi/Levator Nasolabialis Muscle**

This muscle is attached to the frontal and nasal bones. The deep section is attached to the upper lip and lateral wing of the nostril while the superficial section blends with the orbicularis oris muscle in the commissure of the lower lips [[1](#_ENREF_1), [2](#_ENREF_2)] by common tendon with the levator labii superioris proprius muscle. It lies immediately deep to the skin [[1](#_ENREF_1)], although splits and also has a deep section. In contrast to previous references, we found this muscle to be thick and robust [[1](#_ENREF_1)].

**The lower face**

**Transverse Nasi Muscle**

The transverse nasi muscle consists of transverse fibres and attaches to the alar cartilage. It was a very substantial muscle; a cross section taken was over 1cm thick. There were two sections, one that lay deep to the orbicularis oris and the lateralis nasi muscles, and one that lies superficial to the orbicularis oris muscle. This muscle was not reported in [[2](#_ENREF_2)] and has no alternative name in the proposed standardised nomenclature, possibly due to its rarity among mammals [[8](#_ENREF_8)].

**Zygomaticus Muscle**

The zygomaticus muscle lies immediately deep to the skin of the cheek. It is a thin muscle, although clearly defined and thicker than the malaris or the platysma muscles in the horse. The zygomaticus muscle is attached to the fascia covering the masseter muscle below the facial crest, and to the corner of the lips, blending with the buccinator muscle [[1](#_ENREF_1), [2](#_ENREF_2)].

It is generally reported that the horse only has one zygomaticus muscle. However, here we suggest that as in many primates the horse does have both a zygomatic major and a zygomatic minor muscle, and this previous misapprehension has been a result of inconsistent use of nomenclature. We propose that the zygomaticus muscle described here is equivalent to the zygomatic major muscle, and the malaris muscle described below is equivalent to the zygomatic minor muscle.

**Levator Annuli Oris Fascialis Muscle (Also Called The Caninus Or Dilator Naris Lateralis)**

AMB reflected one head of the nasolabialis muscle so that we could see the full extent of the levator annuli oris fascialis muscle going to the base of the nostril. The levator annuli oris fascialis muscle was superficial and lay directly below the skin. It passed between the two branches of the levator nasolabials muscle [[1](#_ENREF_1), [2](#_ENREF_2)], and lies superficial to the buccinator muscle. The levator annuli oris fascialis muscle originated in the rostral end of the facial crest and inserted into the lateral border of the nostril, with its lower fibres blending with the orbicularis oris muscle. The levator annuli oris fascialis muscle has previously been described as thin [[1](#_ENREF_1)], however we found this muscle to be large, thick, and robust, particularly compared to previous primates dissected [[3-6](#_ENREF_3)]. In line with this we found that the levator annuli oris facialis muscle also went into a large and thick tendon.

**Depressor Labii Inferioris Muscle**

This muscle lies on the lateral surface of the mandible along the ventral border of the buccinator muscle [[1](#_ENREF_1), [2](#_ENREF_2)] and extends back along the mid-face to the masseter muscle. This was a large, smooth muscle with longitudinal fibres that attach to the lower lip with a tendon that spreads out, blending with the orbicularis oris and the depressor labii inferioris muscles of the opposite side [[1](#_ENREF_1), [2](#_ENREF_2)].

**Mentalis Muscle**

The mentalis muscle is small and located deep in the prominence of the mental region, ventral to the orbicularis oris muscle and rostral to the buccinator muscle. The fibres of the mentalis muscle arise from each side of the mandible and are inserted into the skin of the [[1](#_ENREF_1)], running in an opposing direction to the fibres of the buccinator muscle. The buccinator and the mentalis muscles are separated by a fascial cleft. The mentalis muscle was omitted from one source that we referenced [[2](#_ENREF_2)], and we found it to be more distinct than previously described [[1](#_ENREF_1)].

**Orbicularis Oris Muscle**

This is a large, thick, and complex sphincter muscle that covers much of the lower face and extends well beyond the external corner of the lips, as far back as the rostral border of the masseter muscle. We also found a very large tendon in the orbicularis oris muscle that was not seen in the facial muscles of any of the other previous primates dissected [[3-6](#_ENREF_3)], and have not found evidence of in other ruminants [[1](#_ENREF_1)]. Tendons allow a greater specificity of movement and strengthen the muscle, in addition to being important in connecting muscle chains, so this tendon seems to support the complex muscle mass of the lower face in the horse.

The orbicularis oris muscle is much larger and thicker than seen in previous primate dissections including the chimpanzee, which was previously reported to have an unusually large orbicularis oris muscle. A cross section taken from the upper lip was approximately 3cm thick, the caudal section from near the masseter muscle was approximately 2cm thick, and a cross section taken from the lower lip was approximately 1cm thick.

The orbicularis oris muscle contains the incisivus superior and inferior. These have previously been recorded as separate muscles [[1](#_ENREF_1)] although here we consider them regions of specialization of the orbicularis oris, rather than specific muscles, as has been done previously in primates [[7](#_ENREF_7)]. The orbicularis oris muscle is attached to numerous other facial muscles, including the buccinator, the dilator nares, and the transverse nasi muscles.

**Malaris Muscle**

This muscle is very thin and lies directly below the skin, superficial to the zygomaticus muscle. Sisson [[1](#_ENREF_1)] describes it as being variable across subjects (as it is in humans, chimpanzees, and rhesus macaques [[5](#_ENREF_5), [6](#_ENREF_6), [9](#_ENREF_9)]), although we found it to be present and distinct in our specimen, and it has also been described in other sources [[2](#_ENREF_2)]. The malaris muscle originates in the dorsal part of the facial crest blending with the skin posterior to the modiolus, and inserts into the lower eyelid [[2](#_ENREF_2)].

The malaris muscle is a term commonly used to describe the zygomatic muscles of other species. The malaris muscle in the horse is seen to follow the same path as the zygomatic minor muscle in humans and other primates (stretching from the skin posterior to the corner of the mouth and blending into the orbicularis oculi muscle, as in humans)[[5](#_ENREF_5), [6](#_ENREF_6)]. Therefore, we propose that the malaris muscle described here is equivalent to what is commonly termed the zygomatic minor muscle in primates.

**The ear**

The external ear comprises of the scutiform cartilage and the auricle (also called the pinna or conchal). The muscles of the ear were large and well defined. There were a number of clear, distinct muscles twirling around the ear, and forming windmill like shape around the scutiform cartilage. As the head was disarticulated from the neck of the atlanto-occipital joint the cervical portion of the skin was missing. This meant we were missing some muscles in the cervical region, and could not report the attachments of other muscles with confidence. Additionally, the instrinsic muscles (the anti tragicus and the helicis) were not investigated as these are very small muscles that are confided to the auricle [[1](#_ENREF_1)], and are not important in moving the external ear.

The muscles of the ear were arranged in a complex, multi-layered fashion, with no evidence of the muscles acting like a sheet, but that the different muscles were clearly differentiated. We also found a large fat pad at the base of the ear, and a large amount of adipose tissue surrounding the auricular muscles.

**Scutularis Muscle**

This is a thin muscular sheet situated subcutaneously over the temporalis muscle. Its fibres arise from the zygomatic arch and the frontal and parietal crests, and converge to the scutiform cartilage [[1](#_ENREF_1)], which was a useful landmark for identification purposes. The scutularis muscle is divided into three: the interscutularis, the frontoscutularis, and the cervio-scutularis muscles.

**Frontoscutularis Muscle**

This is further divided into two parts, the temporal and frontal parts. The temporal part arises from the zygomatic arch and the frontal part arises from the frontal crest. Both insert into the anterior border of the scutiform cartilage [[1](#_ENREF_1), [2](#_ENREF_2)]. This was a thin band of muscle with fibres running longitudinally.

**Interscutularis Muscle (Frontalis Muscle)**

The interscutularis muscle arises from the parietal crest, over which it is partially continuous with the muscle from the other side, and converges with the medial part of the scutiform cartilage [[1](#_ENREF_1), [2](#_ENREF_2)]. This was a thin, flat sheet of muscle with transverse fibres. This muscle termed the frontalis muscle in primates, and seems to be a relatively similar size to that seen in other primates [[3-6](#_ENREF_3)].

**Cervicoscutularis Muscle**

This was not well defined from the interscutularis muscle, and was also a thin flat sheet of muscle with transverse fibres. It arises from the nuchal crest and inserts into the medial border of the scutiform cartilage [[1](#_ENREF_1)].

**The Anterior Auricular Muscles**

**Scutulo-auricularis Superficialis Muscle**

This muscle arises on the scutiform cartilage and ends in the base of the conchal cartilage with the zygomatico-auricularis, the cervicoscutularis and the interscutularis muscles [[1](#_ENREF_1)]. The scutulo-auricularis superficialis is listed as one muscle in some references, e.g. [[2](#_ENREF_2)] but divided into three parts that are considered separately in others e.g. [[1](#_ENREF_1)]. However, in order to keep the scutulo-auriculartis profundus major and minor muscles intact we could not get a detailed view of the scutulo-auricularis superficialis muscles to examine this.

**Zygomatico-auricularis Muscle**

This is a thin, broad sheet of muscle with lateral fibres that arise from the zygomatic arch and the parotid fascia, and insert ventro-medially on the auricle, partly above and partly below the insertion of the partido-auricularis muscle [[1](#_ENREF_1), [2](#_ENREF_2)].

**The Dorso Auricular Muscles**

**Scutulo-auricularis Superficialis Accesorius Muscle**

The scutulo-auricularis superficialis accesorius muscle is a narrow band that arises from the posterior scutiform cartilage and the adjacent part of the superficial face of the scutiform cartilage, and is inserted into the surface of the conchal cartilage medial to the scutuloauricularis superior muscle with the two crossing each other at an acute angle [[1](#_ENREF_1)]. This muscle was not reported in Budras et al. [[2](#_ENREF_2)].

**Scutulo-auriculartis Profundus Major And Minor Muscles**

These muscles are very deep, distinct, thick band that arise from the scutiform cartilage and insert into the auricle. The scutulo-auricularis profudus major muscle has been described as the strongest of the ear muscles [[1](#_ENREF_1)], and we agree with this statement. It is flat and approximately 2.5cm wide.

**Tragicus Muscle**

The tragicus muscle is a very small muscle that arises from the temporal bone just behind the acoustic process, and from the anular cartilage. It passes upwards to be inserted into the lower part of the anterior border of the conchar cartilage [[1](#_ENREF_1)]. This muscle is not reported in Budras et al., [[2](#_ENREF_2)] however we found it present as described in [[1](#_ENREF_1)].

**The Posterior Auricular Muscles**

The head used for this dissection was disarticulated from the neck of the atlantooccipital joint and we were missing the cervical region of the neck. Consequently, we could not confidently label the posterior auricular muscles. This included the cervico-auricularis superficialis, and the cervico auricularis profundus major and minor muscles [[1](#_ENREF_1), [2](#_ENREF_2)].

References

1. Sisson S, Grossman JD. The Anatomy of the Domestic Animals. 4th ed. Philadelphia and London: W. B. Saunders company; 1962.

2. Budras KD, Sack WO, Rock S, Horowitz A, Berg R. Anatomy of the Horse: with Aaron Horowitz and Rolf Berg. 6th ed. Hanover, Germany: Schlutersche Verlagsgesellschaft mbH & Co. KG; 2012.

3. Burrows AM, Diogo R, Waller BM, Bonar CJ, Liebal K. Evolution of the Muscles of Facial Expression in a Monogamous Ape: Evaluating the Relative Influences of Ecological and Phylogenetic Factors in Hylobatids. The Anatomical Record: Advances in Integrative Anatomy and Evolutionary Biology. 2011;294(4):645-63. doi: 10.1002/ar.21355.

4. Burrows AM, Smith TD. Muscles of facial expression in Otolemur, with a comparison to lemuroidea. Anat Rec A Discov Mol Cell Evol Biol. 2003;274(1):827-36. Epub 2003/08/19. doi: 10.1002/ar.a.10093. PubMed PMID: 12923893.

5. Burrows AM, Waller BM, Parr LA. Facial musculature in the rhesus macaque (Macaca mulatta): evolutionary and functional contexts with comparisons to chimpanzees and humans. Journal of Anatomy. 2009;215(3):320-34. doi: 10.1111/j.1469-7580.2009.01113.x.

6. Burrows AM, Waller BM, Parr LA, Bonar CJ. Muscles of facial expression in the chimpanzee (Pan troglodytes): descriptive, comparative and phylogenetic contexts. Journal of Anatomy. 2006;208(2):153-67. doi: 10.1111/j.1469-7580.2006.00523.x.

7. Diogo R, Wood BA. Comparative Anatomy and Phylogeny of Primate Muscles and Human Evolution: Science Publishers; 2012.

8. Diogo R, Wood BA, Aziz MA, Burrows A. On the origin, homologies and evolution of primate facial muscles, with a particular focus on hominoids and a suggested unifying nomenclature for the facial muscles of the Mammalia. Journal of Anatomy. 2009;215(3):300-19. Epub 2009/06/18. doi: 10.1111/j.1469-7580.2009.01111.x. PubMed PMID: 19531159; PubMed Central PMCID: PMC2750763.

9. Waller BM, Cray JJ, Burrows AM. Selection for universal facial emotion. Emotion. 2008;8(3):435-9. Epub 2008/06/11. doi: 10.1037/1528-3542.8.3.435. PubMed PMID: 18540761.
