# Supplementary material for: EquiFACS: The Equine Facial Action Coding System
Source: PLoS One. 2015 Aug 5;10(8):e0131738. doi: 10.1371/journal.pone.0131738 (PMC4526551; doi:10.1371/journal.pone.0131738)
Supplement: S3 Text — (DOCX) [file pone.0131738.s007.docx]

## How to use the EquiFACS manual

The EquiFACS manual lists the identified facial movements, accompanied by a description of the appearance changes and video illustrations. Where necessary, the proposed muscular basis of the actions is given and there are also sections on subtle differences between similar actions. Begin by reviewing these descriptions and video examples. Initially, it might seem impossible to distinguish the facial movements, but just take time to review and reflect on the manual. Additionally, try and understand the muscular basis of the actions. When watching the video examples identify: the parts of the face that have moved and the direction of their movement; any wrinkles that have appeared or deepened; and the alterations in the shape and outline of the face.

Good knowledge of the neutral position of a horse’s face is key. This is true for an individual horse and for horses in general. If you are unfamiliar with horses take some time to first observe a few horses in their relaxed state. See Figure 2 for some key facial landmarks of the horse Figure 3 for a guide to anatomical direction. Be aware that changes in camera position, lighting, and position of the horse’s head can all influence the perception of the face. Be particularly cautious of coding in these situations, and only code an action where there is no doubt that it has occurred. This is especially applicable in non-standard situations, such as those where the horse is rubbing its face against something or chewing. These actions may cause the face to change shape, but not through any contraction of the facial muscles.

It is not necessary to be trained in any of the other FACS systems to be able to learn EquiFACS. However, as with all FACS systems EquiFACS requires certification to use. A test is available from the corresponding authors of the EquiFACS team. If you have any questions about EquiFACS and how you might use it, or would like advice on coding or extra practice materials, please contact Jen Wathan ([j.wathan@sussex.ac.uk](mailto:j.wathan@sussex.ac.uk)).

## Scoring techniques

When scoring a clip make an initial assessment of the action based on gut instinct and record this. Then go back and re-watch the clip, mark any queries you might have on the scoring sheet (e.g. AU 10 or 122?), and refer to the manual to help you resolve these. Review the clip and each part of the face (upper, ears, and lower) to see if you have omitted any possible actions, and check the miscellaneous codes and gross behaviours. Decide on a final score and list the AUs and ADs in numerical order.

Facial expressions are fluid and flexible behaviours. Your specific research question will determine exactly what to code. Generally expressions are coded as discrete movements, and only the apex of an action is coded (the point of maximum expression). Actions are not counted as new events unless there is a complete return to neutral in between (e.g. in AU122, the upper lip curls and then the lips press together – a new action would be counted if the upper lip then curled again). When facial actions are slight it can be difficult to establish exactly which AU occurred. It can be that although the movement may by barely noticeable, there is still enough evidence for you to decide that a particular AU is present. However, if this is not the case then do not code any action.

With all animals it is likely that situations will occur where part of the face is obscured, and there are codes to denote these situations (see S5 Text). However, whereas with primates, dogs, and cats, the most useful information is captured from a frontal view, with horses the best position is an intermediate (3/4) view between frontal and profile. Consequently, it is likely that in most coding situations some information from one side of the face will be missing (the ears are an exception here, as both ears and their movements can usually be seen clearly regardless of the viewpoint). The visibility codes are not needed for these situations, but rather are used to reflect cases where the face is obscured in a way that influences coding, for example if the forelock is covering the horse’s eyes.

We have not yet systematically investigated how symmetrical facial movements of horses are. However, from our observations it seems that a large number of facial movements are generally bilateral (AUH13 is an exception). For that reason, unless specifically interested in symmetry or laterality of facial expressions, we suggest coding movements as bilateral unless evidence of unilaterality can be clearly seen. Additionally, be cautious when coding any unilateral movements, as movements are only truly unilateral when there is absolutely no sign of the movement on the other side of the face. If there is even a trace of movement on the other side of the face, movements must be scored as bilateral. An ‘L’ or ‘R’, always coded from the perspective of the horse, can be used to prefix AUs that are genuinely unilateral.

If specifically interested in symmetry/laterality then it will be necessary to use two video cameras so that one camera can capture movements on each side of the face. Visibility codes (S5 Text) can be used to denote which areas of the face are visible in each view, and which are not.
