# Supplementary material for: EquiFACS: The Equine Facial Action Coding System
Source: PLoS One. 2015 Aug 5;10(8):e0131738. doi: 10.1371/journal.pone.0131738 (PMC4526551; doi:10.1371/journal.pone.0131738)
Supplement: S4 Text — (DOCX) [file pone.0131738.s008.docx]

**Calculating the percentage of eye-white visible (optional)**

This method follows one used previously in cows [[1-5](#_ENREF_1)]. The percentage of white of the total visible eye area can be calculated by measuring the area of the whole eye (T) and the area of the iris (including the pupil - I), then subtracting T from I, dividing this by T, and multiplying this value by 100 [(T-I/T) x100].

The most accurate way of doing this is to use computer software, such as SigmaScanPro, that calculates the area of a selected region. However, as this software is not available to all, a less accurate, but still effective, way is to treat the shape of the eye as an ellipse and calculate T and I by measuring the two orthogonal diameters of T and I, then plugging the values into the formula below. These measurements can be made by simply placing a ruler on the screen, or more accurately by drawing lines on the image in image manipulating software. They can be taken at time points to suit the specific research question and information required.

**A**

**B**

Formula for calculating the area of an ellipse:

Area = **πAB**

The two areas can then be calculated by using the above formula (total eye: **T**; iris: **I**) and the percentage of white of the total visible eye (**PW**) can then be calculated as **PW** = 100 x ((**T**-**I**)/**T**).

References

1. Sandem AI, Janczak AM, Braastad BO. A short note on effects of exposure to a novel stimulus (umbrella) on behaviour and percentage of eye-white in cows. Applied Animal Behaviour Science. 2004;89(3-4):309-14. doi: 10.1016/j.applanim.2004.06.011.

2. Sandem AI, Braastad BO, Bøe KE. Eye white may indicate emotional state on a frustration-contentedness axis in dairy cows. Applied Animal Behaviour Science. 2002;79(1):1-10. doi: <http://dx.doi.org/10.1016/S0168-1591(02)00029-1>.

3. Sandem AI, Janczak AM, Salte R, Braastad BO. The use of diazepam as a pharmacological validation of eye white as an indicator of emotional state in dairy cows. Applied Animal Behaviour Science. 2006;96(3-4):177-83. doi: 10.1016/j.applanim.2005.06.008.

4. Sandem AI, Braastad BO. Effects of cow–calf separation on visible eye white and behaviour in dairy cows—A brief report. Applied Animal Behaviour Science. 2005;95(3-4):233-9. doi: 10.1016/j.applanim.2005.04.011.

5. Core S, Widowski T, Mason G, Miller S. Eye white percentage as a predictor of temperament in beef cattle. Journal of Animal Science. 2009;87(6):2168-74. Epub 2009/02/14. doi: 10.2527/jas.2008-1554. PubMed PMID: 19213718.
