# Supplementary material for: EquiFACS: The Equine Facial Action Coding System
Source: PLoS One. 2015 Aug 5;10(8):e0131738. doi: 10.1371/journal.pone.0131738 (PMC4526551; doi:10.1371/journal.pone.0131738)
Supplement: S5 Text — (DOCX) [file pone.0131738.s009.docx]

# Miscellaneous Actions and Supplementary Codes

These describe additional facial actions that can influence the facial expression. For this reason, it is strongly recommended that the codes listed here are used. While AUs are identified in terms of their muscular basis, Action Descriptors (ADs) cover broader movements, or movements caused by vascular rather than muscular structures. For this reason, “Section A. Proposed muscular basis” has been omitted below.

## Action Descriptor 19: Tongue Show

**B. Appearance changes:**

1. The tongue is shown and it reaches beyond the teeth.
2. The jaw must be lowered and the lips separated. Consequently, this AD is coded together with AU25+AU26 or AU25+AU27.
3. This often happens with a chewing action (AD81).

## Action Descriptor 29: Jaw Thrust

**B. Appearance changes:**

1. The lower jaw is pushed forward (see S44 Video).
2. The lower teeth extend in front of the upper teeth.

**C. Minimum criteria to code AD29:** the lower jaw moves forward.

## Action Descriptor 30: Jaw Sideways

**B. Appearance changes:**

1. The lower jaw is moved sideways (see S44 Video).
2. Chin and lower lip displaced from the midline to one side or the other.
3. If the mouth is open, the lower teeth appear to be off to one side.
4. If you wish to the direction of jaw movement can be scored, by prefixing the code with an L (for left) or R (for right). Always code direction from the perspective of the horse.

**C. Minimum criteria to code AD30:** lateral displacement of the lower jaw so that it does not lie directly under the upper jaw.

## Action Descriptor 133: Blow

In humans AD33 describes air being blow out through the lips, expanding the cheeks. Horses do a similar action, but blow air out through the nostrils rather than the lips. To represent this difference, this action is labeled AD133 in horses.

**B. Appearance changes:**

1. Air is blown out sharply through the nostrils, often accompanied by an obvious noise (see S45 Video).
2. The skin behind the nostril wing may stretch and expand.
3. This action will cause the nostrils to dilate briefly turning into a rounder shape. However, there is no need to code AD38 unless the nostril dilation lasts longer than the blowing action, or if there is a specific interest in the dilation of the nostrils.

**C. Minimum criteria to code AD133:** air is blown sharply through the nostrils.

## Action Descriptor 38: Nostril Dilator

**B. Appearance changes:**

1. The aperture of the nostril is increased (see S46 and S47 Videos).
2. The nostril wings are flared and may bulge as the vascular network in the nare fills with blood.
3. The septum may be lifted and flared, and again may bulge (S47 Video).
4. The shape of the nostril opening may change.
5. The nostril wings may be pushed forward.

**C. Minimum criteria to code AD38:** an increase in the aperture of the nostril.

## Gross Behaviour Codes

**Action Descriptor 50** – Vocalization

**Action Descriptor 76** - Yawning

**Action Descriptor 80** – Swallow

**Action Descriptor 81** – Chewing

**Action Descriptor 84** – Head shake side to side

**Action Descriptor 85** – Head nod up and down

**Action Descriptor 86 –** Grooming

**Action Descriptor 87** – Ear shake

**Head Movement Codes**

Changes in head position can influence the perception of the face, and may also contribute to the overall facial expression: for this reason head movement codes (ADs 51 – 55) are available to denote these changes. These codes should be applied to distinct shifts in orientation from a neutral position, where the horse has the head aligned with the body.

In previous FACS systems the ideal viewing perspective to code facial movements is from directly in front of the animal at face level, and so these codes were applicable from the coder’s viewpoint. However, this is not always the ideal situation when coding the facial movements of horses. Often a perspective that is intermediate between frontal and profile views (3/4 view) is ideal, as this allows the coder to see the complex movements of the lip whilst also able to see movements of the nostrils and eyes. However, still try and use the codes to denote a change from the neutral position, even if not coding from that perspective.

N.B. Always code directions from the perspective of the subject being coded.

Action Descriptor 51: Head Turn Left

The head moves left along a vertical axis.

## Action Descriptor 52: Head Turn Right

The head moves right along a vertical axis.

##

## Action Descriptor 53: Head Up

The head moves upwards.

## Action Descriptor 54: Head Down

The head moves downwards.

## Action Descriptor 55: Head Tilt Left

The head is tilted to the left side.

## Action Descriptor 56: Head Tilt Right

The head is tilted to the right side.

##

## Action Descriptor 57: Nose Forward

The nose is pushed forward, and this is not due to a head toss.

## Action Descriptor 58: Nose Back

The nose is brought in towards the chest.

**Eye Movement Codes**

The extent to which horses can move their eyes to focus on stimuli is still unknown. For this reason, the eye movement codes 61-64 are not used in EquiFACS.

## Visibility Codes

These codes provide information about what areas of the face are not visible and able to be scored. In practical research situations, the face is often obscured, for example by the viewing angle, other animals, or the environment. These codes are a way to handle such situations. These codes can be used to distinguish times when no FACS codes are being scored because nothing is happening from no scores because the face or a part of the face cannot be seen.

Do not score an area of the face as “not visible” if it is possible to score any AU that affects the referenced area. Scores can be applied to only the left or the right side of the face if particularly interested in asymmetry by prefixing the code with L or R.

**Visibility Code 70: Frontal Region Not Visible**

The brow and frontal region (excluding the ears) cannot be seen.

**Visibility Code 71: Eyes Not Visible**

The eyes cannot be seen.

**Visibility Code 72: Lower Face Not Visible**

The lower face cannot be seen.

**Visibility Code 73: Entire Face Not Visible**

The entire face is either out of view or cannot be clearly seen.

**Visibility Code 74: Unscorable**

This code is used when it appears there is something to code but for some reason (e.g. image out of focus) the movements cannot be clearly identified. This code is for the entire facial area and not for unilateral problems with visibility.

**Visibility Code 75: Ears Not Visible**

The ears cannot be seen. This can be suffixed by an L or an R to identify if only one ear is out of sight.
